# Haemato-immunological responses and effectiveness of feed-based bivalent vaccine against *Streptococcus iniae* and *Aeromonas hydrophila* infections in hybrid red tilapia (*Oreochromis mossambicus × O. niloticus*)

**DOI:** 10.1186/s12917-020-02443-y

**Published:** 2020-07-02

**Authors:** Md Shirajum Monir, Sabri bin Mohd Yusoff, Zarirah binti Mohamed Zulperi, Hasliza binti Abu Hassim, Aslah Mohamad, Muhamad Sofie bin Mohd Hafiz Ngoo, Md Yasin Ina-Salwany

**Affiliations:** 1 Department of Aquaculture, Faculty of Agriculture, Universiti Putra Malaysia (UPM), 43400 Serdang, Selangor Malaysia; 2 Department of Veterinary Pathology and Microbiology, Faculty of Veterinary Medicine, Universiti Putra Malaysia (UPM), 43400 Serdang, Selangor Malaysia; 3 Department of Veterinary Preclinical Sciences, Faculty of Veterinary Medicine, Universiti Putra Malaysia (UPM), 43400 Serdang, Selangor Malaysia; 4 Laboratory of Marine Biotechnology, Institute of Bioscience, Universiti Putra Malaysia (UPM), 43400 Serdang, Selangor Malaysia

**Keywords:** Haemato-immunological parameters, Feed-based, Bivalent vaccine, Hybrid red tilapia (*Oreochromis mossambicus × O. niloticus*)

## Abstract

**Background:**

Streptococcosis and Motile Aeromonad Septicemia (MAS) are important diseases of tilapia, *Oreochromis* spp. and causes huge economic losses in aquaculture globally. The feed-based vaccination may be an alternative to minimize major infectious diseases in tilapia. Thus, this study aims to evaluate the haemato-immunological responses and effectiveness of a newly developed feed-based killed bivalent vaccine against *Streptococcus iniae* and *Aeromonas hydrophila* in hybrid red tilapia. A total of 495 hybrid red tilapia of 61.23 ± 4.95 g were distributed into 5 groups (each with triplicate). The fish were immunized orally through bivalent (combined *S. iniae* and *A. hydrophila*) spray vaccine (BS group), bivalent formulate vaccine (BF group), monovalent *S. iniae* vaccine (MS group), monovalent *A. hydrophila* vaccine (MA group) and unvaccinated as a control group. The vaccine was orally administered on days 0, 14 and 42 applied feed-based bacterin at 5% body weight. The blood and spleen samples were collected from all groups on 7, 21 and 49 days post-vaccination, and also 96 h post-infection to assess their haemato-immune responses.

**Results:**

Compared with the unvaccinated group, leukocyte, lymphocytes, monocytes, granulocytes counts in vaccinated groups were significantly (*P* < 0.05) increased on 21, 49 days post-vaccination and also 96 h post-infection, while erythrocytes, haemoglobin and haematocrit in vaccinated groups were significantly (*P* < 0.05) enhanced only 96 h post-infection. Additionally, the lysozyme and phagocytic activity and, serum antibody (IgM) were significantly higher (*P* < 0.05) against *S. iniae* and *A. hydrophila* in vaccinated groups compared to the unvaccinated group in the pre- and post-infection. Results from the challenge through co-infection with *S. iniae* and *A. hydrophila* showed the relative percent survival (RPS) in BF group was 76.67 ± 4.71%, which had the capacity to induce significant protection (*P* < 0.05) compared to others groups.

**Conclusions:**

This study demonstrates the bivalent formulate (BF) group could elicit significant non-specific and specific immunological responses with higher protection in hybrid red tilapia. In addition, this newly developed feed-based bivalent vaccination can be a promising technique for effective and large scale fish immunization in the aquaculture industry.

## Background

According to FAO [1], aquaculture is a fast-growing industry playing an important role in the food producing sector, and has provided good quality and affordable protein source throughout the world [2]. Due to rapid growth, suitability for aquaculture, high acceptability in the market and stable market prices, tilapia (*Oreochromis* spp.) is the most important farmed fish globally next to carps, and is cultured more than 100 countries [3, 4]. The global production of tilapia was calculated around 6.532 million tons in 2018 [5, 6] and is presumed to reach 7.3 million tons by 2030 [7].

Amongst different important bacterial diseases of tilapia, Streptococcosis and Motile Aeromonad Septicaemia (MAS) are the major obstruction to the sustainable development of tilapia farming mainly in Asia [8, 9]. Streptococcosis due to *Streptococcus iniae* and *S. agalactiae* is considered one of the key tilapia disease with high morbidity and mortality throughout the world including Malaysia [10]. It was calculated globally that the yearly economic loss due to Streptococcosis outbreaks was as high as $150 million [11]. On the other hand, another bacterial disease of MAS mainly by *Aeromonas hydrophila* known to have a great negative impact on the growth with survival rate and significant economic losses in the tilapia industry worldwide [12]. Additionally, mass mortality in tilapia due to bacterial co-infection have also been recorded, such as co-infection of *Aeromonas* sp. and *Streptococcus* sp*.* [8, 13], *S. agalactiae* and *F. noatunensis* [14], *A. veronii* and *F. columnare* [15] and *F. noatunensis* subsp. *orientalis* and *Shewanella putrefaciens* [16].

To address the losses due to Streptococcosis and MAS, antibiotics are the only option for farmers and the use of antibiotics results in development of antibiotic resistance strains, bio-accumulation, changes the physio-chemical properties of water, imbalance of bacterial microbiota in fish body or in the habitat [17, 18]. To solve these difficulties, application of fish vaccines is an alternative to minimize the different infectious bacterial diseases [19].

Vaccines are formulated using either antigens, developed from pathogenic bacterial, or whole bacterial killed cells, which improve the specific immunity of the host [20]. Generally, antigens enhance to activate the innate and the adaptive immune systems, both with cellular and humoral responses. The effectiveness of a good vaccine is related to the appropriate immunization routes for stimulating the fish immune system, with advantages and disadvantages. Among the different immunization routes, intraperitoneal (i.p.) injection has mostly provided good results, although it is stressful to the animals, impractical for farmers level, labour intensive and hence expensive [21, 22]. However, another route of immunization is recently practised; the feed-based oral immunization since it is less tedious, more applicable for mass vaccination at farmers level. This immunization route has also proven efficacy in poultry and mammals, which is associated with enhancing of both mucosal and systemic immune systems [23, 24]. Nevertheless, there is still complexity to understand the mechanisms of oral vaccination that are involved in the uptake of antigens. Although, results from oral immunization in fish are contradictory but several researches showed satisfactory results [25, 26]. Kahieshesfandiari [27] reported a positive findings with using feed-based biofilm vaccine in tilapia after challenge against *S. agalactiae*. According to Nur-Nazifah [28], red tilapia immunized orally which presented 70% RPS after *S. agalactiae* challenge.

The application of *A. hydrophila* vaccine is not commercially available for its bio-chemical heterogeneity and the presence of different isolates or sero-groups and variation in virulence gene expression [29, 30]. Nevertheless, very few findings have proved that different vaccine formulations of *A. hydrophila* may provide protection. Besides on, Aly [31] developed an inactivated *A. hydrophila* vaccine for tilapia and after challenge the RPS was satisfactory. Pasaribu [9] prepared an effective bivalent vaccine for tilapia brood stock with formalin inactivated *S. agalactiae* and *A. hydrophila,* and the RPS after challenge by *A. hydrophila* was 73.81%.

As the diversity of infectious bacterial agents is very high intensity in the tilapia culture or production systems, bivalent or multivalent vaccines can be conferred the advantage of protection to tilapia against a wide variety of Gram-negative and Gram-positive bacterial strains [32]. Nevertheless, to date from our knowledge, there is no available study on haemato-immunological responses and protection in hybrid red tilapia immunized with feed-based formalin killed bivalent vaccine against Streptococcosis and MAS. Hence, this study aimed to assess the influence of a feed-based formalin killed bivalent *S. iniae* and *A. hydrophila* vaccine on the haematological and immunological parameters and, protective efficacy in immunized tilapia against challenge with *S. iniae* and *A. hydrophila* each bacterium, independently or co-infection.

## Results

### Haematological parameters

On 7 days post-vaccination the levels of erythrocytes, thrombocytes, lymphocytes, monocytes, granulocytes and haemoglobin were not statistically different (*P* > 0.05) among the groups, but only the number of leucocytes in immunized groups were significantly higher (*P* < 0.05) than the unvaccinated group (Table 1). After 21 days post-immunization, the leucocytes count, monocytes and granulocytes were significantly higher in vaccinated groups than in the unvaccinated, and those from the BF group presented significantly the highest (*P* < 0.05) leucocytes (45.39 ± 1.34 × 10^3^/μl) and granulocytes values (7.19 ± 0.23 × 10^3^/μl) (Table 1). In this study, the number of leucocytes, lymphocytes, monocytes and granulocytes were significantly increased (*P* < 0.05) in all the vaccinated groups at 49 days post-vaccination compared with the unvaccinated group. In addition, leucocytes (47.45 ± 3.22 × 10^3^/μl) and monocytes count (0.55 ± 0.06 × 10^3^/μl) were the highest in MS group, while the level of granulocytes (8.09 ± 0.71 × 10^3^/μl) was the highest in BF group (*P* < 0.05) at 49 days post-vaccination (Table 1). However, no significant (*P* > 0.05) differences of MCH, MCHC and haematocrit had been observed in different vaccinated groups on 7, 21 and 49 days post-vaccination compared to unvaccinated group (Table 2).

**Table 1:** Haematological parameters (mean ± SD) of hybrid red tilapia on days 7, 21 and 49 post-vaccination, before challenge trial

Groups	Days after	Erythrocytes (10^6^/μl)	Thrombocytes (10^3^/μl)	Leucocytes (10^3^/μl)	Lymphocytes (10^3^/μl)	Monocytes (10^3^/μl)	Granulocytes (10^3^/μl)	Haemoglobin (g/dl)
Unvaccinated	7 (dpv)	4.22 ± 0.55^a^	22.43 ± 3.33^a^	26.71 ± 4.51^c^	25.49 ± 1.95^a^	0.30 ± 0.07^a^	4.76 ± 0.46^a^	5.45 ± 1.18^a^
BS		4.98 ± 1.51^a^	23.37 ± 3.59^a^	31.90 ± 1.92^b^	26.35 ± 2.59^a^	0.33 ± 0.05^a^	4.52 ± 0.73^a^	5.38 ± 1.47^a^
BF		5.51 ± 1.96^a^	24.49 ± 3.91^a^	37.23 ± 0.46^a^	28.46 ± 5.71^a^	0.36 ± 0.05^a^	5.45 ± 1.66^a^	5.80 ± 1.49^a^
MS		5.22 ± 2.14^a^	25.56 ± 6.35^a^	36.46 ± 1.20^a^	27.43 ± 5.65^a^	0.35 ± 0.07^a^	5.30 ± 1.13^a^	5.82 ± 1.30^a^
MA		4.46 ± 0.86^a^	24.13 ± 3.92^a^	35.20 ± 0.63^a,b^	27.26 ± 3.89^a^	0.33 ± 0.06^a^	4.90 ± 0.20^a^	5.69 ± 1.49^a^
Unvaccinated	21 (dpv)	3.92 ± 1.52^a^	19.90 ± 3.10^a^	29.21 ± 4.83^c^	17.82 ± 1.52^c^	0.37 ± 0.06^b^	4.29 ± 0.47^c^	4.90 ± 1.84^a^
BS		5.93 ± 2.50^a^	21.77 ± 2.96^a^	37.03 ± 2.08^b^	24.39 ± 1.10^c^	0.39 ± 0.10^a,b^	5.71 ± 1.15^b^	4.98 ± 1.04^a^
BF		7.55 ± 2.06^a^	25.34 ± 5.06^a^	45.39 ± 1.34^a^	27.63 ± 1.40^a,b^	0.46 ± 0.03^a,b^	7.19 ± 0.23^a^	6.58 ± 1.55^a^
MS		6.36 ± 2.66^a^	27.30 ± 4.99^a^	38.75 ± 2.00^b^	28.65 ± 2.84^a^	0.49 ± 0.03^a^	5.69 ± 0.55^b^	6.71 ± 2.31^a^
MA	49 (dpv)	8.01 ± 2.02^a^	23.31 ± 4.89^a^	39.78 ± 2.49^b^	25.93 ± 1.44^a,b^	0.43 ± 0.02^a,b^	5.73 ± 0.40^b^	6.35 ± 1.05^a^
Unvaccinated		4.22 ± 0.94^a^	17.80 ± 5.13^a^	27.92 ± 1.59^c^	18.66 ± 2.84^c^	0.39 ± 0.02^b^	3.96 ± 0.60^c^	5.11 ± 0.66^a^
BS		4.64 ± 1.58^a^	23.62 ± 5.37^a^	39.35 ± 0.86^b^	23.57 ± 1.95^b^	0.49 ± 0.02^a,b^	5.74 ± 0.31^b^	5.73 ± 2.12^a^
BF		6.52 ± 2.10^a^	26.55 ± 5.26^a^	43.18 ± 2.14^b^	27.65 ± 1.88^a,b^	0.52 ± 0.09^a,b^	8.09 ± 0.71^a^	6.79 ± 2.13^a^
MS		7.14 ± 1.51^a^	24.83 ± 6.25^a^	47.45 ± 3.22^a^	28.80 ± 1.73^a^	0.55 ± 0.06^a^	6.16 ± 0.69^b^	7.01 ± 2.66^a^
MA		6.35 ± 2.32^a^	21.11 ± 3.11^a^	42.19 ± 2.99^b^	27.10 ± 2.15^a,b^	0.53 ± 0.03^a,b^	6.11 ± 0.63^b^	6.57 ± 2.26^a^

^a-c^Means within the same column shows a significantly different effect (*P* < 0.05)

**Table 2:** Haematological and immunological parameters (mean ± SD) of hybrid red tilapia on days 7, 21 and 49 post-vaccination, before challenge

Groups	Days after	Haematological parameters			Immunological parameters		
		MCH (pg)	MCHC (g/dl)	Haematocrit (%)	Lysozyme activity (units/ml)	Phagocytic activity (%)	Antibody level (IgM) (450 nm)
Unvaccinated	7 (dpv)	14.24 ± 2.24^a^	29.43 ± 2.94^a^	17.32 ± 4.21^a^	93.73 ± 2.08^d^	23.41 ± 2.03^c^	0.23 ± 0.02^a^
BS		13.75 ± 5.32^a^	31.38 ± 3.82^a^	17.94 ± 5.26^a^	158.35 ± 9.62^c^	27.74 ± 1.52^b^	0.27 ± 0.06^a^
BF		13.90 ± 4.22^a^	28.96 ± 5.89^a^	19.71 ± 4.97^a^	212.77 ± 8.98^a^	32.00 ± 1.40^a^	0.27 ± 0.04^a^
MS		14.12 ± 3.96^a^	30.63 ± 6.67^a^	18.52 ± 2.74^a^	202.17 ± 4.08^a,b^	32.10 ± 1.51^a^	0.28 ± 0.03^a^
MA		13.24 ± 4.87^a^	29.91 ± 6.65^a^	18.72 ± 5.25^a^	192.96 ± 5.50^b^	30.84 ± 1.35^a^	0.26 ± 0.05^a^
Unvaccinated	21 (dpv)	16.71 ± 2.07^a^	38.58 ± 3.15^a^	17.79 ± 2.24^a^	93.60 ± 2.80^d^	24.21 ± 3.54^b^	0.25 ± 0.03^c^
BS		15.95 ± 1.52^a^	39.28 ± 1.74^a^	18.58 ± 4.01^a^	204.16 ± 9.10^a,b^	34.55 ± 2.53^a^	0.40 ± 0.08^b^
BF		14.98 ± 3.23^a^	40.91 ± 3.28^a^	20.17 ± 5.85^a^	264.78 ± 6.99^a^	38.27 ± 4.12^a^	0.62 ± 0.02^a^
MS		16.63 ± 2.48^a^	43.99 ± 4.86^a^	19.44 ± 3.80^a^	253.68 ± 6.78^a,b^	39.67 ± 6.51^a^	0.49 ± 0.09^b^
MA		13.68 ± 1.45^a^	40.92 ± 3.80^a^	17.85 ± 6.67^a^	242.55 ± 6.26^c^	36.28 ± 4.32^a^	0.50 ± 0.03^b^
Unvaccinated	49 (dpv)	17.10 ± 3.01^a^	36.27 ± 2.17^a^	16.49 ± 4.41^a^	94.75 ± 4.43^d^	23.66 ± 3.92^b^	0.21 ± 0.04^c^
BS		16.97 ± 4.10^a^	38.39 ± 2.47^a^	18.86 ± 2.75^a^	207.84 ± 9.43^c^	33.17 ± 5.10^a,b^	0.58 ± 0.03^b^
BF		15.93 ± 4.16^a^	40.75 ± 4.29^a^	19.69 ± 4.39^a^	325.90 ± 6.02^a^	40.88 ± 5.02^a^	0.78 ± 0.02^a^
MS		17.01 ± 4.43^a^	40.13 ± 6.58^a^	17.86 ± 2.04^a^	313.34 ± 5.19^a,b^	39.63 ± 7.81^a^	0.71 ± 0.01^a^
MA		15.92 ± 4.05^a^	43.45 ± 5.83^a^	17.92 ± 3.27^a^	303.05 ± 8.57^b^	38.92 ± 7.22^a^	0.63 ± 0.03^a,b^

^a-d^Means within the same column shows a significantly different effect (*P* < 0.05)

All the haematological parameters of the vaccinated groups, including erythrocytes, leucocytes, lymphocytes, monocytes, granulocytes and haemoglobin were significantly (*P* < 0.0) increased in both *S. iniae* (Table 3) and *A. hydrophila* (Table 4) infection. However, the percentage of haematocrit increased significantly (*P* < 0.05) in the immunized groups, but there were no significant differences in MCH and MCHC levels among the vaccinated and unvaccinated fish following infection with both *S. iniae* (Table 5) and *A. hydrophila* (Table 6).

**Table 3:** Haematological parameters (mean ± SD) after 96 h challenged with *S. iniae* of hybrid red tilapia

Groups	Hours after	Erythrocytes (10^6^/μl)	Thrombocytes (10^3^/μl)	Leucocytes (10^3^/μl)	Lymphocytes (10^3^/μl)	Monocytes (10^3^/μl)	Granulocytes (10^3^/μl)	Haemoglobin (g/dl)
Unvaccinated	96 (hpi)	2.98 ± 0.29^b^	15.87 ± 4.17^a^	18.70 ± 4.50^c^	21.55 ± 3.57^b^	0.24 ± 0.04^b^	3.34 ± 0.51^b^	3.97 ± 1.64^b^
BS		5.19 ± 0.55^a^	20.01 ± 6.21^a^	24.02 ± 1.74^b,c^	27.67 ± 1.00^a^	0.30 ± 0.03^a^	4.97 ± 0.62^a,b^	5.87 ± 0.55^a^
BF		6.9 ± 0.99^a^	22.76 ± 3.63^a^	33.19 ± 2.48^a^	29.50 ± 1.10^a^	0.34 ± 0.15^a^	6.24 ± 1.48^a^	6.25 ± 0.56^a^
MS		7.02 ± 1.81^a^	24.63 ± 3.84^a^	29.03 ± 4.57^a^	30.17 ± 2.10^a^	0.33 ± 0.30^a^	6.06 ± 0.53^a^	7.02 ± 0.75^a^

^a-c^Means within the same column shows a significantly different effect (*P* < 0.05)

**Table 4:** Haematological parameters (mean ± SD) after 96 h challenged with *A. hydrophila* in hybrid red tilapia

Groups	Hours after	Erythrocytes (10^6^/μl)	Thrombocytes (10^3^/μl)	Leucocytes (10^3^/μl)	Lymphocytes (10^3^/μl)	Monocytes (10^3^/μl)	Granulocytes (10^3^/μl)	Haemoglobin (g/dl)
Unvaccinated	96 (hpi)	2.36 ± 0.78^b^	14.15 ± 1.64^a^	19.76 ± 1.95^c^	23.36 ± 4.43^b^	0.26 ± 0.03^b^	2.96 ± 1.10^b^	3.60 ± 0.62^b^
BS		4.54 ± 0.99^a^	18.90 ± 4.28^a^	23.93 ± 1.34^b^	29.55 ± 1.26^a^	0.31 ± 0.03^a^	5.47 ± 0.69^a^	5.86 ± 0.43^a^
BF		5.91 ± 0.67^a^	19.38 ± 3.04^a^	28.10 ± 1.75^a^	30.52 ± 1.93^a^	0.33 ± 0.02^a^	6.10 ± 0.77^a^	6.54 ± 0.97^a^
MA		5.36 ± 1.63^a^	20.13 ± 4.81^a^	25.93 ± 1.56^a,b^	29.08 ± 2.64^a^	0.34 ± 0.02^a^	5.99 ± 0.68^a^	5.95 ± 0.68^a^

^a-c^Means within the same column shows a significantly different effect (*P* < 0.05)

**Table 5:** Haematological and immunological parameters (mean ± SD) after 96 h challenged with *S. iniae* of hybrid red tilapia

Groups	Hours after	Haematological parameters			Immunological parameters		
		MCH (pg)	MCHC (g/dl)	Haematocrit (%)	Lysozyme activity (unit/ml)	Phagocytic activity (%)	Antibody level (IgM) (450 nm)
Unvaccinated	96 (hpi)	15.58 ± 2.82^a^	21.99 ± 1.38^a^	13.45 ± 2.71^b^	96.50 ± 4.16^c^	27.45 ± 6.14^c^	0.30 ± 0.02^c^
BS		13.08 ± 2.26^a^	28.79 ± 3.32^a^	18.45 ± 2.78^a^	253.37 ± 6.11^b^	36.50 ± 3.49^b^	0.59 ± 0.06^b^
BF		12.90 ± 5.03^a^	29.46 ± 7.82^a^	20.26 ± 1.57^a^	327.83 ± 6.38^a^	47.60 ± 4.50^a^	0.86 ± 0.07^a^
MS		14.79 ± 2.20^a^	30.29 ± 7.83^a^	21.06 ± 2.65^a^	323.57 ± 3.04^a^	39.00 ± 9.05^a,b^	0.72 ± 0.03^a,b^

^a-c^Means within the same column shows a significantly different effect (*P* < 0.05)

**Table 6:** Haematological and immunological parameters (mean ± SD) after 96 h challenged with *A. hydrophila* in hybrid red tilapia

Groups	Hours after	Haematological parameters			Immunological parameters		
		MCH (pg)	MCHC (g/dl)	Haematocrit (%)	Lysozyme activity (unit/ml)	Phagocytic activity (%)	Antibody level (IgM) (450 nm)
Unvaccinated	96 (hpi)	15.17 ± 0.65^a^	20.00 ± 1.75^a^	12.85 ± 1.39^b^	97.84 ± 4.24^d^	26.39 ± 5.50^b^	0.30 ± 0.01^c^
BS		14.75 ± 0.81^a^	23.15 ± 3.79^a^	18.38 ± 1.01^a^	235.54 ± 6.84^c^	37.15 ± 2.96^a,b^	0.53 ± 0.08^b^
BF		15.36 ± 2.05^a^	19.38 ± 3.04^a^	19.45 ± 1.12^a^	323.07 ± 4.59^a^	48.04 ± 6.61^a^	0.75 ± 0.04^a^
MA		14.97 ± 1.52^a^	22.67 ± 3.03^a^	16.59 ± 2.72^a^	301.03 ± 5.41^b^	47.07 ± 7.45^a^	0.65 ± 0.04^a^

^a-d^Means within the same column shows a significantly different effect (*P* < 0.05)

### Immunological parameters

The value of lysozyme activity and phagocytic activity showed a statistically different effect (*P* < 0.05) between the groups of vaccinated and unvaccinated after 7 days post-immunization, but the antibody level (IgM) did not show statistically difference (*P* > 0.05) among the groups. On day 21 post-vaccination, the value of lysozyme activity, phagocytic activity and antibody level (IgM) were significantly higher in vaccinated groups than in the unvaccinated group, and those from the BF (0.62 ± 0.02) group presented the highest antibody level against *S. iniae* (*P* < 0.05) among the different groups. At 49 days post-vaccination, both lysozyme activity and phagocytic activity were significantly higher in vaccinated groups compared to the unvaccinated group, whereas BF group also showed the statistically highest (*P* < 0.05) lysozyme (325.90 ± 6.02 units/ml) activity comparisons with other groups. On the other hand, the antibody (IgM) level of vaccinated groups were also significantly higher against *S. iniae* and *A. hydrophila* (*P* < 0.05) in comparison to the unvaccinated group, whereas the BF (0.78 ± 0.02), MS (0.71 ± 0.01) and MA (0.63 ± 0.03) groups were obtained significantly the highest level among the groups at 49 days post-vaccination (Table 2).

After challenged with *S. iniae*, the unvaccinated fish group presented significantly lower (*P* < 0.05) lysozyme activity, phagocytic activity and antibody level compared to the vaccinated group. Additionally, the lysozyme activity was significantly (*P* < 0.05) higher in both BF (327.83 ± 6.38 units/ml) and MS (323.57 ± 3.04 units/ml) groups, while only the BF group showed the highest phagocytic activity (47.60 ± 4.50%) and antibody level (0.86 ± 0.07) when compared with others groups (Table 5). Furthermore, it was observed that after challenged with *A. hydrophila,* the value of lysozyme activity, phagocytic activity and antibody level (IgM) showed a significant (*P* < 0.05) difference compared to the unvaccinated group. Consequently, the antibody level was also significantly higher (*P* < 0.05) in both BF (0.75 ± 0.04) and MA (0.65 ± 0.04) groups, followed by BS (0.53 ± 0.08) and unvaccinated group (0.30 ± 0.01) (Table 6) and (Additional file 1).

### Mortality and relative percentage of survival (RPS)

**Fig. 1:**
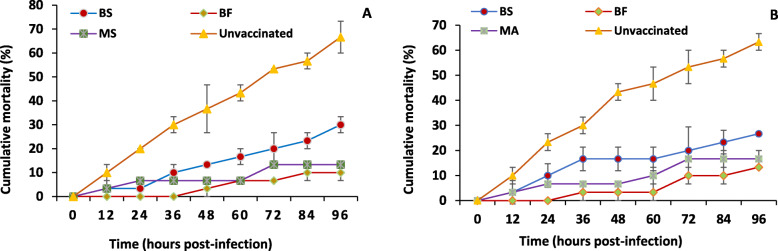
Cumulative mortalities (%) of hybrid red tilapia after challenged with *Streptococcus iniae* (**a**) and *Aeromonas hydrophila* (**b**)

**Fig. 2:**
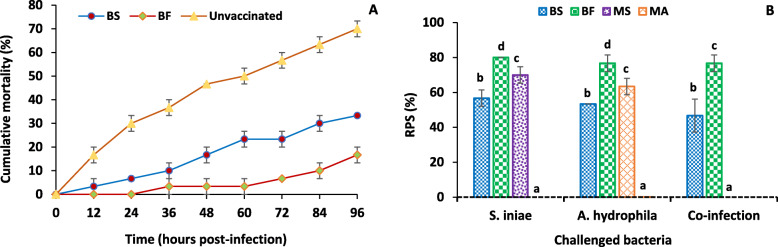
Cumulative mortalities (%) of hybrid red tilapia after challenged with co-infection (**a**), and RPS (%) of hybrid red tilapia (**b**). The fish were challenged on post-vaccination days 70, and monitored for up to14 days after post-infection

Percent cumulative mortalities up to 96 h after infection with *S. iniae* and *A. hydrophila* or co-infection in vaccinated and unvaccinated groups are shown in Fig. 1a, b and Fig. 2a. After challenge (i.p. injection) with *S iniae*, the cumulative mortality percentage was statistically (*P* < 0.05) higher in the unvaccinated group than in the vaccinated groups, and those from the BF (10 ± 4.71%) and MS (13.33 ± 0.00%) groups at 96 h post-infection presented the lowest cumulative mortality against *S. iniae* (*P* < 0.05) among the different groups (Fig. 1a). Similar results were obtained in the challenge with *A. hydrophila*, where the cumulative mortality percentage was also significantly lower (*P* < 0.05) in the groups of BF (13.33 ± 0.00%) and MA (16.67 ± 4.72%) compared with the unvaccinated group (Fig. 1b). Consequently, the cumulative mortality of the unvaccinated group (70.00 ± 4.71%) showed significantly higher (*P* < 0.05) than other bivalent groups after co-infection with both *S. iniae* and *A. hydrophila*. (Fig. 2a). However, 100% mortalities were observed only in unvaccinated group after 168 h post-infection with *S. iniae* and *A. hydrophila* or co-infection with both the bacteria (Additional file 2)*.*

Following 14 days post-infection with *S. iniae*, the RPS was significantly higher (*P* < 0.05) in BF (80.00 ± 0.00%) and MS (70.00 ± 4.71%) groups compared with the BS group (56.67 ± 4.71%). Furthermore, after challenged with *A. hydrophila*, the RPS was also obtained significantly higher protection in BF (76.67 ± 4.71%) and MA (63.33 ± 4.71%) when compared with the group of BS (53.33 ± 0.00%) (Fig. 2b). Simultaneously, under the challenge of co-infection with both *S iniae* and *A. hydrophila,* BF group produced significantly higher RPS (76.67 ± 4.71%), indicating that bivalent vaccine candidate of BF group could confer much better protection against *S iniae* and *A. hydrophila* compared to the other immunized groups.

## Discussion

Haematological parameters have been usually used for observing the health status and immunological responses of fish and aquatic animals. Among the different haematological parameters, the leukocyte count is very important for functioning in the non-specific and specific immune system of the fish [33]. In this study, the leukocyte count in all the vaccinated group was higher than unvaccinated group, and those from the BF vaccinated fish presented the highest number of leukocyte on 7 and 21 days post-vaccination and post-infections. Bailone [34] observed that vaccinated tilapia had higher leukocyte count compared to the unvaccinated fish both before and after challenge with *A. hydrophila.* Furthermore, the leukocyte count in immunized sturgeon (*Huso huso*) were higher against *A. hydrophila* [35]. According to Ashfaq [36], the increase in leukocyte count positively affects antibody production, leading to body resistance response against the extraneous substance. Likewise, Silva [22] established, a higher count of leukocyte was strongly correlated with the increasing of phagocytic activity, lysozyme function and antibody titers. In the present experiment, leukocyte count was higher as a result of vaccine can indirectly increase natural immune response which is marked by an increase in phagocytic cells. On the contrast, it indicates in this study that the immunized fish were capable to show better defence response against infection with *S. iniae* and *A. hydrophila*.

In other assays, the total number of erythrocyte was higher in vaccinated red tilapia compared to unvaccinated fish after challenge. The higher number of erythrocyte counts were in MS and BF vaccinated groups than other vaccinated groups after infection with *S. iniae* and *A. hydrophila*, respectively. However, these findings agree with those recorded by Silva [22] who revealed the erythrocytes count of immunized tilapia with formalin killed vaccine via i.p. injection was higher compared with unvaccinated fish after challenge with *A. hydrophila.* On the other hand, previous findings demonstrated that the decreasing erythrocytes count after challenge in unvaccinated fish may be signs of bacterial infection [37]. Subsequently, previous reports also show that the number of erythrocytes decreased in coho salmon (*Oncorhynchus kisutch*), rainbow trout (*Salmo gairdneri*) after infected with *V. anguillarum*, *Aeromonas* sp*./Streptococcus* sp*.*, respectively [38, 39]. However, in this study, erythrocyte count in the unvaccinated group significantly decreased, this might be due to *A. hydrophila* and *S. iniae* had hemolysin that can cause erythrocyte lysis [40, 41].

Haemoglobin is a pigment in erythrocyte and has a function to bind oxygen to be further circulated to all over the body. Thus, haemoglobin and haematocrit percentage are good signs for the oxygen transportation capacity of fish [42]. In this study, the haemoglobin and haematocrit percentage in vaccinated fish did not show a significant different compared to the unvaccinated fish after immunization. Sukenda [43] also documented that the haemoglobin and haematocrit percentage of vaccinated tilapia were not significantly different from unimmunized fish. Once again, both haemoglobin and haematocrit level of immunized tilapia was increased following infection with *S. iniae* and *A. hydrophila*, which was also observed in immunized Nile tilapia after infection with *A. hydrophila* [44]. These results indicate that the low protective effect of the immune system in unvaccinated fish, which led *S. iniae* or *A. hydrophila* to actively release erythrocytes and further reduced haemoglobin levels in blood after challenges. Nevertheless, these findings also indicates that there was no anemic states develop in feed-based immunized fish after challenged with both bacteria.

Monocytes/macrophage in both mammals and fishes are a vital component of the mononuclear phagocytic systems, and play significant role during infections, inflammation, tissue injuries and repairs [45]. In the current study, the number of monocytes and granulocytes were significantly increased in the vaccinated tilapia on 21, 49 days post-vaccination and after infection with *S. iniae* and *A. hydrophila* as well. In addition, only on 21 and 49 days post-vaccination, the monocytes counts showed higher *(P* > 0.05) in both BF and MS than other vaccinated groups. However, Chin and Woo [46] observed that vaccinated salmon (*S. salar*) with live *Cryptobia salmositica* showed a significant increase in the number of monocytes and granulocytes on days 28 and 42 post-immunization, respectively. Moreover, Garcia [47] reported that the number of neutrophils and monocytes in *Piaractus mesopotamicus* were increased after infection with *A. hydrophila.* Nevertheless, increased number of monocytes might be related with a stimulate immune response and phagocytic activity [47].

Lymphocytes counts did not differ significantly on 7 days post-vaccination, but an increase in the lymphocyte counts was observed only in vaccinated tilapia on 21, 49 days post-vaccination and also after infection with both bacteria. The lymphocytes counts was significantly higher in the vaccinated *Ictalurus punctatus,* fish [48], which was similar to the current findings. Sirimanapong [49] showed that the number of lymphocyte was also significantly higher in the vaccinated fish by 1, 14 and 21 days post-immunization. In contrast, Pereira [19] reported that the lymphocytes counts were not statistically different after oral vaccination in surubim hybrid (*Pseudoplatystoma corruscans* x *P. reticulatum*). However, the neutrophils and monocytes in the current study played a significant role in the early immune response followed by the lymphocytes. In the current study, there was non significant (*P* > 0.05) increase of thrombocytes in both pre and post infection in the vaccinated groups relative to the unvaccinated group. Pereira [19] also did not find significant changes of thrombocytes counts after vaccination in surubim hybrid (*P. corruscans* x *P. reticulatum*) against haemorrhagic septicaemia. Nevertheless, this is indicated that thrombocytes that is not response to both the vaccination and infection.

Lysozyme is a bacteriolytic enzyme produced in the lysosome of the phagocytic cell and lysozyme activity is an essential part of the non-specific immune response of fish. In this study, vaccinated tilapia showed a significant increase of lysozyme activity compared to the unvaccinated fish in both pre- and post-infection. Additionally, on 7, 49 days post-vaccination and post-infection with *A. hydrophila*, lysozyme activity were significantly higher in fish vaccinated by BF, followed by MA vaccinations. Subsequently, previous reports show that the lysozymes activity was significantly higher in vaccinated than unvaccinated tilapia on 3 weeks post-vaccination [49]. In contrast, Pereira [25] reported, the lysozyme activity did not show a significantly different effect after post-vaccination compared with unvaccinated. Likewise, Sukenda [50] observed that vaccinated broodstock tilapia delivered a significant lysozyme activity in broodstock, eggs, and fry compared to the unvaccinated and showed significant survival in immunized fry. However, this indicates that the increasing reaction of the immune response system in vaccinated tilapia might be correlated with increasing of lysozyme activity.

Fish phagocytes, acting as accessory cells with adaptive immune function, also play a significant role in the innate immune system and are attached in combatting pathogen infection at all steps. The phagocytic cells are functioned in stimulating an inflammatory response, phagocytosis and bactericidal action and nitric oxide (NO) for killed pathogens. In the current study, the phagocytosis activity in vaccinated fish was higher both before and after infection with *S. iniae* and *A. hydrophila* compared with the unvaccinated fish, which was similar to record by Kordon [51]. Additionally, similar results have been also shown in vaccinated striped catfish (*Pangasianodon hypophthalmus*) those were immunised with *A. hydrophila* [49]. However, these findings indicate that the peripheral blood of vaccinated fish have phagocytic activity and therefore might also play significant role in the early innate immune response.

Antibody level is the major parameter to evaluate the specific immune response. The findings of this experiment demonstrate that the antibody level (IgM) of the monovalent and bivalent vaccinated tilapia was increased significantly than the unvaccinated fish when tested for both bacterial antigens. Furthermore, antibody level (IgM) of fish vaccinated with BF were higher significantly on days 14, whereas the BF and MS groups were significantly higher than the BS vaccinated fish (*P* < 0.05) only on days 49. In line with this, Nur-Nazifah [28] observed that tilapia vaccinated with the feed-based vaccine improved a strong and significantly higher antibody response in blood serum samples compared to the unvaccinated fish. SongLin [52] also showed that tilapia vaccinated with the bivalent *V. vulnificus* and *A. hydrophila* vaccine and the vaccine delivered a significantly higher level of antibodies against both the antigens. However, this finding revealed that feed-based monovalent or bivalent vaccine can develop protective specific immune responses in hybrid red tilapia.

The low percent of cumulative mortality in vaccinated fish are usually caused due to the development of the specific immune response that followed by an increase of non-specific immune system. In our present work, significantly lower percent cumulative mortality was in vaccinated fish compared with unvaccinated. In some previous experiments were used *Bacillus subtilis* spores expressing sip and *S. agalactiae* ghost, and confirmed their effectiveness in preventing mortalities in immunized tilapia [53, 54]. A similar low mortality rate was documented in rainbow trout immunized with bivalent formalin-inactivated whole cells *A. hydrophila* [50]. However, our findings are also in concordance with other report, in which the use of bivalent vaccine provided a very low mortalities against *A. hydrophila* and *L. garvieae* infections in vaccinated rainbow trout (*Oncorhynchus mykiss*) [55].

Protections levels in the current study obtained in fish immunized with bivalent formulate (BF) group specially those obtained at 70 days post-vaccination (80.00 ± 0.00% and 76.67 ± 4.71% against *S. inaie* and *A. hydrophila* respectively) were higher than those documented for American eel (*Anguilla rostrata*) using oral bivalent vaccine against *V. vulnificus* and *A. hydrophila* as a vaccine antigen of American eel (*Anguilla rostrata*) [52]. In contrast, our findings are in concordance with other results, in which the use of bivalent vaccine provided a successfully lengthen the protections against *L. garvieae* and *A. hydrophila* in rainbow trout (*O. mykiss*) [55]. Yao [53] and Wang [54] observed that the increase of antibody in fish strongly co-related to the survival rate or RPS of fish. However, several works also confirm that the application of feed-based monovalent vaccination in fish increases the RPS or survival significantly, as in tilapia immunized against *S. agalactiae* [27, 28].

The RPS of the BS group in this study was significantly lower compared with the BF group after challenged with co-infection (*S. iniae* and *A. hydrophila*). For bivalent spray (BS) vaccine, commercial pellet feed was directly top-dressed with bivalent FKCs suspension, whereas the formulate bivalent or monovalent vaccines was developed by incorporating or mixing the FKCs on feed powder and finally loaded into the feed pellet machine to make formulated vaccinated feed. Eventually, spraying or top dressing of antigens suspension on pellet feed is quite easy to apply but have the disadvantage of irregular distribution, leaching the antigens during feeding time and also the threat that the antigens are directly exposed to hostile stomach environment upon feeding, leading to degradation [56]. By contrast, mixing or incorporating the antigens with the fish feeds give the advantages of regular distribution of the antigens inside the vaccine pellets feed, and also protecting the antigens against the hostile stomach environment for impregnating antigens inside the feeds. Furthermore, the feed-based formulated vaccine in the current examination brought about higher RPS following infection with both *S. iniae* and *A. hydrophila* in comparison with the consequences of Ismail [25], where fishes had been immunized with top-dressed feed-based oral vaccine. However, considering about these outcome, it could be presumed that formulated or incorporated oral vaccine in the present study has more effective and ensuring better protection of the immunized fish against *S. iniae* and *A. hydrophila* in comparison with top-dressed oral vaccination.

The success of a bivalent or multivalent vaccine is often controlled by the amount of individual antigens, cross reactivity and competition between or among different antigens. In the current study, the protection achieved and the antibody (IgM) response to *S. iniae* and *A. hydrophila* showed the lack of antigenic cross reactivity and competition between these pathogenic bacteria. Bastardo [55] showed that rainbow trout (*O. mykiss*) presented higher immune responses and survival after the immunization of bivalent vaccines, even better than the monovalent vaccines. As a result, throughout the findings further suggest that the bivalent formulate (BF group) vaccine is capable of inducing protective immunity against *S. iniae, A. hydrophila* or co-infection. Nevertheless, in combination with the haematological parameters, immunological responses and protection results in this study, we considered that this was due to the activation of innate and specific immunity after feed-based bivalent immunization.

## Conclusion

Immunization of hybrid red tilapia with a feed-based bivalent formulate (BF) vaccine was demonstrated to confer significant level of strong protections (compared to monovalent vaccines) against *S. iniae* and *A. hydrophila*, as well as co-infection with both bacterial pathogens. In addition, this study also indicates certain positive correlation between protections efficacies and presence of high level of specific antibody and partly relate to different haematological parameters, phagocytic and lysozyme activities. The use of this vaccine formulation may provide cost-effective strategies to minimize losses in hybrid red tilapia co-infected with *S. iniae* and *A. hydrophila* bacteria. These results also suggest that feed-based oral bivalent vaccination can be a promising technique for effective and large scale fish immunization in the aquaculture industry.

## Methods

### Fish and experimental conditions

The study was carried out at Fish Hatchery Unit in Laboratory of Marine Biotechnology (MARSLAB), Institute of Bio-Science (IBS), Universiti Putra Malaysia, Malaysia. A total of 605 apparently healthy hybrid red tilapia (*Oreochromis mossambicus × O. niloticus*) with an average weight of 61.23 ± 4.95 g, were purchased from a local fish farm (Kam Sing Fish farm, Selangor, Malaysia). The collected red tilapia were randomly distributed into 18 tanks with 400 l capacity. The fish were acclimatized for 14 days before vaccination and fed with an available commercial diet (Star Feed, Star Feed Mills SDN. BHD, Malaysia) with 32% protein containing feed at 3% body weight per day. The fish faces and waste materials were siphoned out 3 h after feeding. Prior to commence the experiment, 30 fish were randomly dissecting for screening pathogenic bacteria and checking the antibody level to confirm that they were free from *Streptococcus* sp. and *Aeromonas* sp*.* One day prior to vaccination or challenge fish were taken off feed. The water quality of the rearing tanks like temperature, pH, dissolved oxygen, ammonia and nitrites were observed throughout the study period. Anesthetics were applied on the experimental fish using 120 mg/L of tricaine methanesulfonate, MS-222 (Aldrich, USA) prior to collect blood samples and bacterial challenge protocols.

### Formalin-killed bacteria preparation

The pathogenic strains of *S. iniae* and *A. hydrophila* were isolated from diseased hybrid red tilapia and obtained from the previous study [57, 58]. The formalin killed bacterin were prepared as stated in the previous studies [25, 28]. Briefly, the bacterial strains of *S. iniae* and *A. hydrophila* were cultured individually on 5% blood agar (Oxoid, UK) and further grown in separate flasks of 500 ml containing Brain Heart Infusion Broth (BHIB, Oxoid, UK) at 30 °C in a shaker incubator at 150 rpm for overnight. The following incubation, the bacterial concentrations were calculated by applying the establish plate count. The individually cultured bacteria cells were then inactivated by treating with neutral-buffered formalin to the concentration of 0.5% formalin in PBS (phosphate buffered saline) and kept at 4 °C for 24 h. After that, the bacterial cells were washed four times with the sterile PBS by centrifugation at 6000 x g for 15 min to remove the medium and formalin residue from the culture. Afterwards, the inactivated bacteria were again suspended in sterile PBS to keep the final concentration of 6.7 × 10^9^ CFU/ml. The bacterial suspension was again streaked onto BHIA and incubated at 37 °C for overnight to confirm that all *S. iniae* or *A. hydrophila* cells were inactivated. For formulations of bivalent vaccine, formalin killed whole cells (FKCs) of two vaccine strains were combined at a ratio of 1:1 (*v/v*) and kept at 4 °C. Subsequently, for improving the vaccine antigenicity, palm oil (Vesawit, Malaysia) as an adjuvant was mixed to a final concentration of 10% before it was thoroughly sprayed on commercial pellet or formulate feed to obtain a final concentration of 6.7 × 10^9^ cells/g of feed [25, 28].

### Feed-based vaccine preparation

#### Bivalent spray vaccine

The feed-based vaccine was formulated according to the method described earlier Ismail [25] with some modifications. Briefly, the formalin-killed bactrin (FKB) of *S. iniae* (6.7 × 10^9^ CFU/ml) and *A. hydrophila* (6.7 × 10^9^ CFU/ml) with 10% palm oil were mixed and re-suspended properly in PBS for preparing bivalent vaccine. Next, the bivalent FKB solution was directly sprayed onto the commercial floating pellet feed (Star feed, containing 32% protein) to obtain the individual FKB a final concentration of 6.7 × 10^9^ cells/g of feed. A homogenizer or mixer (Golden Bull B10-A Universal Mixer, Malaysia) was used to distribute and impregnate the bivalent FKB vaccine properly into the pellet feed. Finally, the vaccine added pellet was dried up at 30 °C for overnight in the oven prior to the experiment.

#### Preparation of formulated vaccine

A commercially available pellet feed (Star feed, containing 32% protein) was blended with a blender machine to form a very fine mesh powder. To incorporate the vaccine, the formalin-killed bacterin (FKB) of monovalent or bivalent vaccine with 10% palm oil was re-suspended in PBS to a final concentration of 6.7 × 10^9^ CFU/ml. Afterwards, the FKB solution of monovalent or bivalent vaccine was sprayed properly onto the fish feed powder to obtain the individual FKB a final concentration of 6.7 × 10^9^ cells/g of feed. A homogenizer was used to distribute and impregnate the monovalent or bivalent FKB vaccine properly onto the fish feed powder. In unvaccinated group, only 10% palm oil was added in fish feed as a control group. Finally, the vaccine added feed paste was loaded into the auto mini pellet machine (Golden Avill, China) to make the pellet size of 4 × 4 mm and kept at 30 °C for overnight in the oven prior to the feed-based immunization.

#### Experimental design

**Fig. 3:**
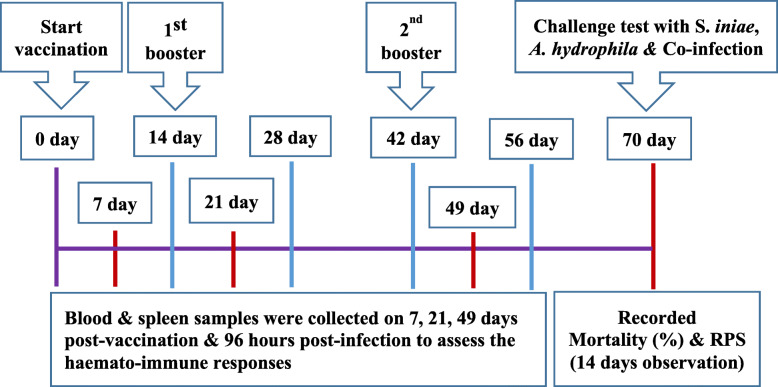
Timeline of vaccination regime and challenge assay

A total of 495 hybrid red tilapia (*Oreochromis mossambicus × O. niloticus*) were randomly distributed into 15 glass aquaria with 400 l capacity. The experiment was conducted with five different experimental major groups, and each group consisted of 99 fish for the 3 replicates; each replicate containing 33 fish in 400 L glass aquaria. Group-1 (unvaccinated) was fed non-vaccine containing commercial pellet feed (incorporated only 10% palm oil); group-2 (Bivalent Spray, BS) was vaccinated by bivalent mixture of *S. iniae* and *A. hydrophila* vaccine directly sprayed on commercial pellet feed; group-3 (Bivalent Formulate, BF) was vaccinated by bivalent vaccine incorporated in feed; group-4 (Monovalent *S. iniae*, MS) was vaccinated by only monovalent *S. iniae* vaccine incorporated in feed and group-5 (Monovalent *A. hydrophila,* MA) was vaccinated by only monovalent *A. hydrophila* vaccine incorporated in feed. At the start of the vaccination, the feed-based vaccine was orally applied in all vaccination groups only on day 0 at 5% body weight four times daily up to 5 consecutive days. Except for unvaccinated control group, all other groups were double boosted with the same immunization on 14 and 42 days after first dose vaccination (Fig. 3). The water was dechlorinated and aerated continuously throughout the trials. The water quality of the experimental glass aquaria were maintained at temperature of (27.73 ± 2.45 °C), dissolved oxygen (6.97 ± 2.43 mg/l), pH (7.65 ± 1.45) and ammonia (0.01 ± 0.00 mg/l) ranged on acceptable levels overall the experimental periods.

#### Challenge test

The challenge tests were performed on 70 days post-vaccination with single bacterial infections of *S. iniae* (3.4 × 10^8^ CFU/ml), *A. hydrophila* (6.8 × 10^9^ CFU/ml) and the co-infection of both pathogenic bacteria (*S. iniae*, 3.4 × 10^8^ CFU/ml and *A. hydrophila*, 6.8 × 10^9^ CFU/ml) with a composition ratio of 1:1. The fish were challenged via intraperitoneal (i.p.) route at a dose of 0.5 ml. Besides the four vaccinated groups, four sub-control groups were set up for challenge test, including control (negative)-1 (unvaccinated, without challenge), control-2 (unvaccinated, but challenged with *S. iniae*) and control-3 (unvaccinated, but challenged with *A. hydrophila*) and control-4 (unvaccinated, but challenged with co-infection, *S. iniae* and *A. hydrophila*). For each bivalent vaccinated group, fish were separated into three sub-groups: The sub-groups were challenged separately with *S. iniae*, *A. hydrophila* and another with co-infection. In every vaccinated group or sub-group was two replicates and each replicate had consisted of 15 fish. All of the unvaccinated or non-challenged each fish was also injected with 0.5 ml PBS. Fish mortalities in each group was subsequently recorded daily for 14 days after the challenges. Following 14 days post-challenges, vaccine efficacies were estimated by comparing the average cumulative mortalities (%) and the relative percentage of survival (RPS: [1- (% mortality in vaccinated fish/ % mortality in unvaccinated fish) × 100]). All remaining fishes at the end of the challenge trials and experiment were euthanized in overdose concentration of 400 mg/L of tricaine methanesulfonate, MS-222 (Aldrich, USA) for at least 10 min and soaked in 25% sodium hypochlorite for 30 min prior disposed as clinical waste.

#### Sample collection

Sampling of fish blood and spleen organ were done from five groups on 7, 21 and 49 days post-vaccination (dpv) and also 96 h post-infection. The blood sample was collected through the caudal veins from 9 fish of each group and the collected blood were kept in two different type tubes, in one EDTA-containing tubes while another without EDTA-containing tubes. The EDTA-containing blood samples were immediately sent to the laboratory for haematological parameters analysis but without EDTA-containing samples were used to assess immune responses. The spleen samples were collected to determine the phagocytic activity.

#### Haematological assays

The anti-coagulate blood samples were used to determine the erythrocyte, thrombocytes, leukocyte, haemoglobin, lymphocytes, haematocrit, MCH (mean corpuscular haemoglobin), MCHC (mean corpuscular haemoglobin concentration), monocytes and granulocytes count using an automatic Mythic 18 Vet Haematology analyser machine (Woodley Veterinary Diagnostics, England and Wales).

### Immunological assays

#### Serum lysozyme activity

The lysozyme assay was done according to the methods of Anderson and Swicki [59]. The pH of the PBS was adjusted to 6.2 at 25 °C using 1 M HCl and 1 M KOH. This (0.01 M PBS) was used to prepare 0.4 mg/ml of *Micrococcus lysodeikticus*. Firstly, 100 μl blood serum and then prepared 100 μl *M. lysodeikticus* was put into the microplate. Afterwards, the resulting absorbance was read at 450 nm (optical density, OD) using microplate reader (Multiskan™ GO Microplate Spectrophotometer, USA) at the time interval of 30 s and after 30 min. The lysozyme activity was calculated based on a decrease in OD of 0.001/min. The following formula was used to estimate the units of enzyme activity per 1 ml of the serum.

$$ \mathrm{Units}/\mathrm{ml}=\frac{\varDelta \mathrm{A}450/\min -\varDelta \mathrm{A}450\min \left(\mathrm{df}\right)}{(0.001)(0.01)} $$

#### Phagocytosis activity

Phagocytosis by spleen performed using the method of Anderson and Swicki [59]. Briefly, in this method; yeast cells were the particulate cells, where equal volumes of spleen cell suspension and yeast cells (0.1 ml) were mixed well with a pipette and incubated for 20 min at 25 °C. Five microliters of the incubated solution was placed on a glass slide (pre-coated with 10% Poly L-Lysine (PLL) solution and dried) and made a smear, air dried and fixed with 95% methanol for 1 min, transferred into May-Grunwald (MG) solution for 5 min. Finally, the cells were stained with 7% Giemsa stain for 20 min, air-dried. The cells were set under oil immersion (100 x magnification) and 100 cells were counted from different portions of the slide and finally, the percentage of phagocytic cells were determined.

#### Enzyme-linked immunosorbent assay (ELISA)

The samples of serum were subjected to indirect ELISA to determine the antibody titre against *S. iniae* and *A. hydrophila* using the method described by Ismail [25] with minor modification. Coating antigen was prepared by culturing *S. iniae* and *A. hydrophila* into BHIB and incubated for overnight in shaker incubator for 150 rpm at 30 °C. The concentration of the cultured bacteria was calculated with the following of the standard plate count method prior to harvest through centrifugation and washed with PBS. After that, the bacterial pellets were suspended in carbonate-bicarbonate buffer (pH 9.6). The prepared bacteria was inactivated through boiling in a water bath at 90 °C for 20 min and incubated at room temperature before to use as coating antigen (2.5 × 10^5^ CFU/ml). Then, 100 μl coating antigen was coated into the microtitre plates and kept at 4 °C for 24 h before washed two times with PBST (PBS + 0.05% Tween 20). This was followed by adding 200 μl of 1% BSA to block unspecific binding sites and kept at 37 °C for 1 hour. Thereafter, 100 μl of diluted serum (1:1000) was added into the reaction and incubated. Afterwards, goat anti-tilapia hyperimmune serum (*Aquatic Diagnostics Ltd*, Scotland) was diluted at the ratio of 1:10000, added 100 μl into the reaction and incubated at 37 °C for 1 hour again. Then, 100 μl of conjugated rabbit anti-goat IgM horseradish peroxidase (Nordic, Netherland), diluted 1:10000 was added and incubated. The following microtitre plates was added 100 μl of TMB (Promega, USA) after washed for thrice with PBST and finally added100 μl of TMB (Promega, USA) before 0.2 mol/l sulphuric acid. The absorbance was calculated by setting microplate reader (Multiskan™ GO Microplate Spectrophotometer, Finland) at 450 nm wavelength.

### Statistical analysis

Data were analysed using SPSS-16 software (SPSS Inc., Chicago IL). Differences in haemato-immunological parameters and RPS between unvaccinated and vaccinated groups were examined using one-way ANOVA with Duncan post hoc tests. Statistical significance was considered at *p* values < 0.05.

## Supplementary information

**Additional file 1: Table 1.** Haemato-immunological parameters in groups wise.

**Additional file 2: Table 2.** Statistics.

## Data Availability

The datasets analysed during the present study are available from the corresponding author on reasonable request.

## Abbreviations

MCH: Mean corpuscular hemoglobin
MCHC: Mean corpuscular hemoglobin concentration;
ELISA: Enzyme-linked immunosorbent assay
IgM: Immunoglobulin M
i.p.: Intraperitoneal
FKB: Formalin killed bactria
dpv: Days post vaccination
hpi: Hours post infection
BS: Bivalent spray vaccine
BF: Bivalent formulate vaccine
MS: Monovalent *S. iniae* vaccine
MA: Monovalent *A. hydrophila* vaccine
