# Supplementary material for: Haemato-immunological responses and effectiveness of feed-based bivalent vaccine against Streptococcus iniae and Aeromonas hydrophila infections in hybrid red tilapia (Oreochromis mossambicus × O. niloticus)
Source: BMC Vet Res. 2020 Jul 2;16:226. doi: 10.1186/s12917-020-02443-y (PMC7330267; doi:10.1186/s12917-020-02443-y)
Supplement: Supplementary file 1 — Additional file 1: Table 1. Haemato-immunological parameters in groups wise. [file 12917_2020_2443_MOESM1_ESM.docx]

| 1. Blood was collected from 3 fish per replication (9 fish/group/timepoint) | **Groups** | | | | |
| --- | --- | --- | --- | --- | --- |
| Sampling time: **7 days post-vaccination** | **Blood parameter (Erythrocytes, 10^6/µl)** | | | | |
| Replication | BS | BF | MS | MA | Unvaccinated |
| R1 (fish-1) | 3.47 | 3.77 | 4.17 | 3.03 | 4.27 |
| R1 (fish-2) | 4.32 | 3.29 | 3.27 | 2.77 | 3.23 |
| R1 (fish-3) | 3.97 | 3.02 | 3.84 | 2.69 | 3.45 |
| Average | 3.92 | 3.36 | 3.76 | 2.83 | 3.65 |
|  |  |  |  |  |  |
| R2 (fish-1) | 3.93 | 6.42 | 7.87 | 7.39 | 5.34 |
| R2 (fish-2) | 3.86 | 5.91 | 7.54 | 6.57 | 5.75 |
| R2 (fish-3) | 3.85 | 6.72 | 7.45 | 6.95 | 5.02 |
| Average | 3.88 | 6.35 | 7.62 | 6.97 | 5.37 |
|  |  |  |  |  |  |
| R3 (fish-1) | 4.71 | 4.32 | 4.88 | 6.14 | 4.54 |
| R3 (fish-2) | 5.03 | 6.03 | 5.05 | 5.63 | 4.31 |
| R3 (fish-3) | 4.81 | 5.37 | 5.52 | 5.78 | 4.26 |
| Average | 4.85 | 5.24 | 5.15 | 5.85 | 4.37 |

**Table 1** Haemto-immunological parameters are showing in groups wise (each group 3 replication and blood collated from 3 fish in each replication) and each time points (7, 21 and 49 days post-vaccination, and 96 hours post-infection with *S. iniae* and *A. hydrophila*).

**Sampling time:** **7 days post-vaccination**

| 1. Blood was collected from 3 fish per replication (9 fish/group/timepoint) | **Groups** | | | | | | | | | |  |
| --- | --- | --- | --- | --- | --- | --- | --- | --- | --- | --- | --- |
| Sampling time: **7 days post-vaccination** | **Blood parameter (Thrombocytes, 10^3/µl** | | | | | | | | | |  |
| Replication | BS | | BF | | MS | | MA | | Unvaccinated | |  |
| R1 (fish-1) | 21.17 | | 20.04 | | 25.37 | | 21.29 | | 23.27 | |  |
| R1 (fish-2) | 20.29 | | 19.47 | | 24.86 | | 20.48 | | 20.16 | |  |
| R1 (fish-3) | 20.25 | | 21.18 | | 24.59 | | 20.24 | | 23.38 | |  |
| Average | 20.57 | | 20.23 | | 24.94 | | 20.67 | | 22.27 | |  |
|  |  | |  | |  | |  | |  | |  |
| R2 (fish-1) | 20.47 | | 23.47 | | 28.63 | | 33.79 | | 28.55 | |  |
| R2 (fish-2) | 20.24 | | 22.92 | | 27.87 | | 32.96 | | 29.88 | |  |
| R2 (fish-3) | 20.64 | | 21.38 | | 27.98 | | 31.47 | | 27.49 | |  |
| Average | 20.45 | | 22.59 | | 28.16 | | 32.74 | | 28.64 | |  |
|  |  | |  | |  | |  | |  | |  |
| R3 (fish-1) | 26.68 | | 28.18 | | 21.47 | | 24.22 | | 23.14 | |  |
| R3 (fish-2) | 26.29 | | 27.48 | | 19.32 | | 22.33 | | 20.18 | |  |
| R3 (fish-3) | 25.87 | | 26.18 | | 20.35 | | 23.23 | | 21.12 | |  |
| Average | 26.28 | | 27.28 | | 20.38 | | 23.26 | | 21.48 | |  |
| 1. Blood was collected from 3 fish per replication (9 fish/group/timepoint) | | **Groups** | | | | | | | | | |
| Sampling time: **7 days post-vaccination** | | **Blood parameter (Leucocytes, 10^3/µl** | | | | | | | | | |
| Replication | | BS | | BF | | MS | | MA | | Unvaccinated | |
| R1 (fish-1) | | 25.48 | | 30.48 | | 36.54 | | 36.45 | | 36.49 | |
| R1 (fish-2) | | 23.35 | | 29.86 | | 37.93 | | 35.13 | | 34.75 | |
| R1 (fish-3) | | 24.85 | | 28.94 | | 35.84 | | 34.23 | | 34.54 | |
| Average | | 24.56 | | 29.76 | | 36.77 | | 35.27 | | 35.26 | |
|  | |  | |  | |  | |  | |  | |
| R2 (fish-1) | | 23.28 | | 34.55 | | 38.69 | | 38.59 | | 35.59 | |
| R2 (fish-2) | | 24.26 | | 33.42 | | 37.75 | | 36.67 | | 36.84 | |
| R2 (fish-3) | | 23.47 | | 32.47 | | 36.63 | | 37.75 | | 34.94 | |
| Average | | 23.67 | | 33.48 | | 37.69 | | 37.67 | | 35.79 | |
|  | |  | |  | |  | |  | |  | |
| R3 (fish-1) | | 33.27 | | 33.14 | | 38.12 | | 35.43 | | 35.18 | |
| R3 (fish-2) | | 30.47 | | 31.12 | | 37.33 | | 37.42 | | 33.22 | |
| R3 (fish-3) | | 31.93 | | 33.12 | | 36.21 | | 36.44 | | 35.22 | |
| Average | | 31.89 | | 32.46 | | 37.22 | | 36.43 | | 34.54 | |

| 1. Blood was collected from 3 fish per replication (9 fish/group/timepoint) | **Groups** | | | | |
| --- | --- | --- | --- | --- | --- |
| Sampling time: **7 days post-vaccination** | **Blood parameter (Lymphocytes, 10^3/µl** | | | | |
| Replication | BS | BF | MS | MA | Unvaccinated |
| R1 (fish-1) | 28.47 | 29.77 | 34.24 | 22.97 | 23.46 |
| R1 (fish-2) | 27.54 | 28.23 | 33.22 | 23.96 | 23.77 |
| R1 (fish-3) | 26.34 | 27.95 | 33.13 | 21.74 | 21.38 |
| Average | 27.45 | 28.65 | 33.53 | 22.89 | 22.87 |
|  |  |  |  |  |  |
| R2 (fish-1) | 24.23 | 23.38 | 23.17 | 34.57 | 29.48 |
| R2 (fish-2) | 23.27 | 24.41 | 23.21 | 32.98 | 29.16 |
| R2 (fish-3) | 23.18 | 22.83 | 20.46 | 33.73 | 27.28 |
| Average | 23.56 | 23.54 | 22.28 | 33.76 | 28.64 |
|  |  |  |  |  |  |
| R3 (fish-1) | 26.54 | 27.93 | 29.84 | 26.43 | 29.28 |
| R3 (fish-2) | 25.46 | 26.83 | 28.66 | 25.94 | 32.43 |
| R3 (fish-3) | 24.38 | 25.85 | 30.24 | 24.58 | 29.13 |
| Average | 25.46 | 26.87 | 29.58 | 25.65 | 30.28 |

| 1. Blood was collected from 3 fish per replication (9 fish/group/timepoint) | **Groups** | | | | |
| --- | --- | --- | --- | --- | --- |
| Sampling time: **7 days post-vaccination** | **Blood parameter (Monocytes, 10^3/µl)** | | | | |
| Replication | BS | BF | MS | MA | Unvaccinated |
| R1 (fish-1) | 0.31 | 0.35 | 0.46 | 0.32 | 0.29 |
| R1 (fish-2) | 0.37 | 0.47 | 0.34 | 0.29 | 0.21 |
| R1 (fish-3) | 0.22 | 0.29 | 0.43 | 0.26 | 0.28 |
| Average | 0.3 | 0.37 | 0.41 | 0.29 | 0.26 |
|  |  |  |  |  |  |
| R2 (fish-1) | 0.36 | 0.29 | 0.42 | 0.48 | 0.39 |
| R2 (fish-2) | 0.39 | 0.31 | 0.32 | 0.38 | 0.41 |
| R2 (fish-3) | 0.36 | 0.24 | 0.22 | 0.43 | 0.34 |
| Average | 0.37 | 0.28 | 0.32 | 0.43 | 0.38 |
|  |  |  |  |  |  |
| R3 (fish-1) | 0.27 | 0.28 | 0.41 | 0.33 | 0.42 |
| R3 (fish-2) | 0.24 | 0.33 | 0.35 | 0.35 | 0.34 |
| R3 (fish-3) | 0.21 | 0.41 | 0.32 | 0.28 | 0.26 |
| Average | 0.24 | 0.34 | 0.36 | 0.32 | 0.34 |

| 1. Blood was collected from 3 fish per replication (9 fish/group/timepoint) | **Groups** | | | | |
| --- | --- | --- | --- | --- | --- |
| Sampling time: **7 days post-vaccination** | **Blood parameter (Granulocytes, 10^3/µl)** | | | | |
| Replication | BS | BF | MS | MA | Unvaccinated |
| R1 (fish-1) | 4.36 | 4.27 | 6.53 | 4.17 | 5.39 |
| R1 (fish-2) | 4.72 | 3.97 | 5.83 | 4.13 | 5.54 |
| R1 (fish-3) | 4.84 | 2.95 | 4.89 | 4.21 | 3.59 |
| Average | 4.64 | 3.73 | 5.75 | 4.17 | 4.84 |
|  |  |  |  |  |  |
| R2 (fish-1) | 3.88 | 5.12 | 7.56 | 7.42 | 5.07 |
| R2 (fish-2) | 5.15 | 5.18 | 6.37 | 5.73 | 5.05 |
| R2 (fish-3) | 4.08 | 5.18 | 6.89 | 6.11 | 5.24 |
| Avergae | 4.37 | 5.16 | 6.94 | 6.42 | 5.12 |
|  |  |  |  |  |  |
| R3 (fish-1) | 5.24 | 4.57 | 4.38 | 6.18 | 5.82 |
| R3 (fish-2) | 4.29 | 5.16 | 2.91 | 5.12 | 3.94 |
| R3 (fish-3) | 6.28 | 4.28 | 3.66 | 4.66 | 4.43 |
| Avergae | 5.27 | 4.67 | 3.65 | 5.32 | 4.73 |

| 1. Blood was collected from 3 fish per replication (9 fish/group/timepoint) | **Groups** | | | | |
| --- | --- | --- | --- | --- | --- |
| Sampling time: **7 days post-vaccination** | **Blood parameter (Haemoglobin, g/dl)** | | | | |
| Replication | BS | BF | MS | MA | Unvaccinated |
| R1 (fish-1) | 5.38 | 4.92 | 6.59 | 7.45 | 8.61 |
| R1 (fish-2) | 4.35 | 3.31 | 5.76 | 6.68 | 6.74 |
| R1 (fish-3) | 3.68 | 2.81 | 5.23 | 6.48 | 6.61 |
| Average | 4.47 | 3.68 | 5.86 | 6.87 | 7.32 |
|  |  |  |  |  |  |
| R2 (fish-1) | 6.08 | 6.98 | 3.59 | 7.18 | 3.57 |
| R2 (fish-2) | 4.15 | 6.02 | 5.12 | 6.13 | 5.49 |
| R2 (fish-3) | 5.13 | 5.57 | 4.13 | 5.32 | 4.05 |
| Average | 5.12 | 6.19 | 4.28 | 6.21 | 4.37 |
|  |  |  |  |  |  |
| R3 (fish-1) | 7.88 | 6.48 | 7.23 | 4.38 | 6.48 |
| R3 (fish-2) | 6.92 | 6.49 | 8.32 | 4.44 | 5.33 |
| R3 (fish-3) | 5.48 | 5.84 | 6.23 | 4.29 | 4.33 |
| Average | 6.76 | 6.27 | 7.26 | 4.37 | 5.38 |

|  | **Groups** | | | | |
| --- | --- | --- | --- | --- | --- |
| 1. Blood was collected from 3 fish per replication (9 fish/group/timepoint) |  |  |  |  |  |
| Sampling time: **7 days post-vaccination** | **Blood parameter (MCH, pg)** | | | | |
| Replication | BS | BF | MS | MA | Unvaccinated |
| R1 (fish-1) | 17.48 | 19.12 | 11.58 | 19.12 | 10.37 |
| R1 (fish-2) | 17.26 | 19.12 | 9.95 | 18.12 | 9.87 |
| R1 (fish-3) | 15.42 | 20.44 | 10.42 | 17.75 | 11.38 |
| Average | 16.72 | 19.56 | 10.65 | 18.33 | 10.54 |
|  |  |  |  |  |  |
| R2 (fish-1) | 13.57 | 10.14 | 19.49 | 11.11 | 19.84 |
| R2 (fish-2) | 15.15 | 8.17 | 17.65 | 9.24 | 18.66 |
| R2 (fish-3) | 12.23 | 9.05 | 18.87 | 11.03 | 17.78 |
| Average | 13.65 | 9.12 | 18.67 | 10.46 | 18.76 |
|  |  |  |  |  |  |
| R3 (fish-1) | 13.27 | 13.45 | 13.45 | 14.55 | 11.11 |
| R3 (fish-2) | 11.37 | 12.59 | 12.27 | 12.59 | 11.82 |
| R3 (fish-3) | 12.44 | 11.67 | 11.45 | 13.57 | 11.36 |
| Average | 12.36 | 12.57 | 12.39 | 13.57 | 11.43 |

| 1. Blood was collected from 3 fish per replication (9 fish/group/timepoint) | **Groups** | | | | |
| --- | --- | --- | --- | --- | --- |
| Sampling time: **7 days post-vaccination** | **Blood parameter (MCHC, g/dl)** | | | | |
| Replication | BS | BF | MS | MA | Unvaccinated |
| R1 (fish-1) | 26.11 | 28.49 | 34.03 | 37.43 | 34.12 |
| R1 (fish-2) | 26.03 | 27.74 | 34.12 | 35.19 | 35.32 |
| R1 (fish-3) | 26.34 | 26.63 | 31.63 | 36.16 | 33.22 |
| Average | 26.16 | 27.62 | 33.26 | 36.26 | 34.22 |
|  |  |  |  |  |  |
| R2 (fish-1) | 32.34 | 32.12 | 23.43 | 24.34 | 22.35 |
| R2 (fish-2) | 32.84 | 31.12 | 22.16 | 22.23 | 23.23 |
| R2 (fish-3) | 30.43 | 30.54 | 21.16 | 23.24 | 21.17 |
| Average | 31.87 | 31.26 | 22.25 | 23.27 | 22.25 |
|  |  |  |  |  |  |
| R3 (fish-1) | 30.22 | 36.23 | 33.56 | 33.45 | 34.23 |
| R3 (fish-2) | 31.29 | 35.31 | 30.69 | 32.28 | 33.12 |
| R3 (fish-3) | 29.27 | 34.24 | 29.83 | 31.35 | 32.43 |
| Average | 30.26 | 35.26 | 31.36 | 32.36 | 33.26 |

| 1. Blood was collected from 3 fish per replication (9 fish/group/timepoint) | **Groups** | | | | |
| --- | --- | --- | --- | --- | --- |
| Sampling time: **7 days post-vaccination** | **Blood parameter (Haematocrit%)** | | | | |
| Replication | BS | BF | MS | MA | Unvaccinated |
| R1 (fish-1) | 15.38 | 15.45 | 18.12 | 16.37 | 25.19 |
| R1 (fish-2) | 16.25 | 14.12 | 17.67 | 16.12 | 23.27 |
| R1 (fish-3) | 14.42 | 13.54 | 16.32 | 15.12 | 24.65 |
| Average | 15.35 | 14.37 | 17.37 | 15.87 | 24.37 |
|  |  |  |  |  |  |
| R2 (fish-1) | 14.55 | 16.88 | 26.84 | 21.76 | 13.64 |
| R2 (fish-2) | 15.38 | 14.84 | 24.93 | 20.14 | 12.88 |
| R2 (fish-3) | 13.45 | 14.66 | 24.49 | 22.12 | 15.42 |
| Avergae | 14.46 | 15.46 | 25.42 | 21.34 | 13.98 |
|  |  |  |  |  |  |
| R3 (fish-1) | 22.33 | 24.54 | 16.44 | 18.63 | 18.45 |
| R3 (fish-2) | 22.03 | 24.19 | 17.24 | 18.93 | 17.64 |
| R3 (fish-3) | 22.12 | 23.21 | 15.34 | 17.52 | 17.34 |
| Avergae | 22.16 | 23.98 | 16.34 | 18.36 | 17.81 |

| 1. Blood was collected from 3 fish per replication (9 fish/group/timepoint) | **Groups** | | | | |
| --- | --- | --- | --- | --- | --- |
| Sampling time: **7 days post-vaccination** | **Immune parameter (Lysozyme, unit/ml)** | | | | |
| Replication | BS | BF | MS | MA | Unvaccinated |
| R1 (fish-1) | 21.14 | 27.91 | 30.84 | 30.68 | 28.25 |
| R1 (fish-2) | 21.23 | 27.81 | 29.57 | 28.94 | 26.23 |
| R1 (fish-3) | 19.34 | 26.72 | 28.84 | 28.76 | 25.23 |
| Average | 20.57 | 27.48 | 29.75 | 29.46 | 26.57 |
|  |  |  |  |  |  |
| R2 (fish-1) | 24.34 | 29.54 | 34.24 | 33.43 | 32.45 |
| R2 (fish-2) | 23.64 | 28.34 | 31.17 | 31.71 | 34.64 |
| R2 (fish-3) | 22.13 | 27.83 | 32.27 | 29.81 | 31.46 |
| Average | 23.37 | 28.57 | 32.56 | 31.65 | 32.85 |
|  |  |  |  |  |  |
| R3 (fish-1) | 25.12 | 28.47 | 29.24 | 32.34 | 28.12 |
| R3 (fish-2) | 25.23 | 26.82 | 28.34 | 34.32 | 27.02 |
| R3 (fish-3) | 23.36 | 27.45 | 30.11 | 33.45 | 30.12 |
| Average | 24.57 | 27.58 | 29.23 | 33.37 | 28.42 |

| 1. Blood was collected from 3 fish per replication (9 fish/group/timepoint) | **Groups** | | | | |
| --- | --- | --- | --- | --- | --- |
| Sampling time: **7 days post-vaccination** | **Immune parameter (Phagocytosis%)** | | | | |
| Replication | BS | BF | MS | MA | Unvaccinated |
| R1 (fish-1) | 24.54 | 27.49 | 34.11 | 30.15 | 32.12 |
| R1 (fish-2) | 23.51 | 25.96 | 33.22 | 31.15 | 32.67 |
| R1 (fish-3) | 22.33 | 25.66 | 32.3 | 30.14 | 32.26 |
| Average | 23.46 | 26.37 | 33.21 | 30.48 | 32.35 |
|  |  |  |  |  |  |
| R2 (fish-1) | 26.34 | 26.54 | 32.26 | 33.14 | 29.45 |
| R2 (fish-2) | 24.55 | 26.95 | 29.67 | 31.17 | 31.33 |
| R2 (fish-3) | 25.37 | 28.98 | 29.48 | 32.74 | 30.45 |
| Average | 25.42 | 27.49 | 30.47 | 32.35 | 30.41 |
|  |  |  |  |  |  |
| R3 (fish-1) | 22.11 | 29.02 | 33.38 | 34.55 | 29.95 |
| R3 (fish-2) | 20.82 | 28.82 | 31.41 | 32.41 | 30.84 |
| R3 (fish-3) | 21.15 | 30.27 | 32.14 | 33.45 | 28.49 |
| Average | 21.36 | 29.37 | 32.31 | 33.47 | 29.76 |

| 1. Blood was collected from 3 fish per replication (9 fish/group/timepoint) | **Groups** | | | | |
| --- | --- | --- | --- | --- | --- |
| Sampling time: **7 days post-vaccination** | **Immune parameter (Antibody level)** | | | | |
| Replication | BS | BF | MS | MA | Unvaccinated |
| R1 (fish-1) | 0.23 | 0.23 | 0.25 | 0.25 | 0.23 |
| R1 (fish-2) | 0.21 | 0.21 | 0.26 | 0.26 | 0.25 |
| R1 (fish-3) | 0.25 | 0.22 | 0.24 | 0.24 | 0.21 |
| Average | 0.23 | 0.22 | 0.25 | 0.25 | 0.23 |
|  |  |  |  |  |  |
| R2 (fish-1) | 0.21 | 0.26 | 0.24 | 0.29 | 0.32 |
| R2 (fish-2) | 0.19 | 0.25 | 0.24 | 0.29 | 0.3 |
| R2 (fish-3) | 0.26 | 0.24 | 0.24 | 0.26 | 0.31 |
| Average | 0.22 | 0.25 | 0.24 | 0.28 | 0.31 |
|  |  |  |  |  |  |
| R3 (fish-1) | 0.23 | 0.34 | 0.32 | 0.31 | 0.21 |
| R3 (fish-2) | 0.26 | 0.36 | 0.29 | 0.27 | 0.21 |
| R3 (fish-3) | 0.26 | 0.29 | 0.32 | 0.32 | 0.27 |
| Average | 0.25 | 0.33 | 0.31 | 0.3 | 0.23 |

**Note:** Bivalent Spray (BS); Bivalent Formulate (BF); Monovalent *S. iniae* (MS); Monovalent *A. hydrophila* (MA). Data were presented in main manuscript **Tables 1 and 2.**

| 2. Blood was collected from 3 fish per replication (9 fish/group/timepoint) | **Groups** | | | | |
| --- | --- | --- | --- | --- | --- |
| Sampling time: **21 days post-vaccination** | **Blood parameter (Erythrocytes, 10^6/µl)** | | | | |
| Replication | BS | BF | MS | MA | Unvaccinated |
| R1 (fish-1) | 4.29 | 7.45 | 9.15 | 5.45 | 7.55 |
| R1 (fish-2) | 3.25 | 6.47 | 7.16 | 6.43 | 5.97 |
| R1 (fish-3) | 5.24 | 4.86 | 8.05 | 4.53 | 5.62 |
| Average | 4.26 | 6.26 | 8.12 | 5.47 | 6.38 |
|  |  |  |  |  |  |
| R2 (fish-1) | 1.93 | 9.53 | 10.23 | 4.26 | 11.44 |
| R2 (fish-2) | 3.43 | 7.59 | 9.23 | 4.18 | 10.74 |
| R2 (fish-3) | 1.42 | 7.63 | 8.32 | 4.34 | 8.63 |
| Average | 2.26 | 8.25 | 9.26 | 4.26 | 10.27 |
|  |  |  |  |  |  |
| R3 (fish-1) | 6.13 | 2.35 | 5.16 | 10.34 | 9.05 |
| R3 (fish-2) | 4.44 | 4.25 | 5.61 | 8.34 | 6.72 |
| R3 (fish-3) | 5.18 | 3.24 | 5.04 | 9.4 | 6.34 |
| Average | 5.25 | 3.28 | 5.27 | 9.36 | 7.37 |

**Sampling time:** **21 days post-vaccination**

| 2. Blood was collected from 3 fish per replication (9 fish/group/timepoint) | **Groups** | | | | |
| --- | --- | --- | --- | --- | --- |
| Sampling time: **21 days post-vaccination** | **Blood parameter (Thrombocytes, 10^3/µl)** | | | | |
| Replication | BS | BF | MS | MA | Unvaccinated |
| R1 (fish-1) | 18.17 | 26.05 | 22.34 | 27.38 | 26.71 |
| R1 (fish-2) | 16.05 | 22.12 | 18.32 | 27.26 | 26.78 |
| R1 (fish-3) | 15.46 | 24.34 | 20.42 | 25.31 | 23.85 |
| Average | 16.56 | 24.17 | 20.36 | 26.65 | 25.78 |
|  |  |  |  |  |  |
| R2 (fish-1) | 19.33 | 19.24 | 31.34 | 33.23 | 28.24 |
| R2 (fish-2) | 22.25 | 20.13 | 31.56 | 32.26 | 26.15 |
| R2 (fish-3) | 19.83 | 16.01 | 28.51 | 32.25 | 25.02 |
| Average | 20.47 | 18.46 | 30.47 | 32.58 | 26.47 |
|  |  |  |  |  |  |
| R3 (fish-1) | 22.85 | 22.28 | 24.06 | 23.65 | 18.87 |
| R3 (fish-2) | 23.25 | 23.89 | 28.05 | 21.82 | 17.78 |
| R3 (fish-3) | 21.94 | 21.84 | 23.43 | 22.54 | 16.39 |
| Average | 22.68 | 22.67 | 25.18 | 22.67 | 17.68 |

| 2. Blood was collected from 3 fish per replication (9 fish/group/timepoint) | **Groups** | | | | |
| --- | --- | --- | --- | --- | --- |
| Sampling time: **21 days post-vaccination** | **Blood parameter (Leucocytes, 10^3/µl** | | | | |
| Replication | BS | BF | MS | MA | Unvaccinated |
| R1 (fish-1) | 35.84 | 37.46 | 45.76 | 38.15 | 40.34 |
| R1 (fish-2) | 36.78 | 32.27 | 42.67 | 34.21 | 37.66 |
| R1 (fish-3) | 31.39 | 34.28 | 43.48 | 37.08 | 36.48 |
| Average | 34.67 | 34.67 | 43.97 | 36.48 | 38.16 |
|  |  |  |  |  |  |
| R2 (fish-1) | 27.36 | 37.65 | 47.56 | 40.29 | 43.87 |
| R2 (fish-2) | 26.66 | 35.83 | 43.54 | 39.15 | 42.72 |
| R2 (fish-3) | 28.36 | 39.98 | 45.58 | 39.12 | 41.36 |
| Average | 27.46 | 37.82 | 45.56 | 39.52 | 42.65 |
|  |  |  |  |  |  |
| R3 (fish-1) | 24.48 | 39.48 | 47.17 | 40.41 | 40.8 |
| R3 (fish-2) | 24.26 | 38.87 | 46.24 | 41.23 | 37.98 |
| R3 (fish-3) | 27.73 | 37.48 | 46.51 | 39.14 | 36.84 |
| Average | 25.49 | 38.61 | 46.64 | 40.26 | 38.54 |

| 2. Blood was collected from 3 fish per replication (9 fish/group/timepoint) | **Groups** | | | | |
| --- | --- | --- | --- | --- | --- |
| Sampling time: **21 days post-vaccination** | **Blood parameter (Lymphocytes, 10^3/µl** | | | | |
| Replication | BS | BF | MS | MA | Unvaccinated |
| R1 (fish-1) | 17.12 | 27.76 | 30.32 | 26.85 | 27.39 |
| R1 (fish-2) | 17.16 | 24.18 | 28.17 | 25.87 | 28.23 |
| R1 (fish-3) | 15.07 | 24.41 | 29.23 | 24.95 | 27.12 |
| Average | 16.45 | 25.45 | 29.24 | 25.89 | 27.58 |
|  |  |  |  |  |  |
| R2 (fish-1) | 18.62 | 24.14 | 27.78 | 33.25 | 26.67 |
| R2 (fish-2) | 17.68 | 22.56 | 26.94 | 32.29 | 25.35 |
| R2 (fish-3) | 16.38 | 23.05 | 25.89 | 29.17 | 22.83 |
| Average | 17.56 | 23.25 | 26.87 | 31.57 | 24.95 |
|  |  |  |  |  |  |
| R3 (fish-1) | 20.56 | 25.46 | 26.41 | 30.12 | 23.49 |
| R3 (fish-2) | 18.48 | 23.83 | 28.45 | 27.14 | 24.97 |
| R3 (fish-3) | 19.34 | 24.12 | 25.48 | 28.18 | 27.32 |
| Average | 19.46 | 24.47 | 26.78 | 28.48 | 25.26 |

| 2. Blood was collected from 3 fish per replication (9 fish/group/timepoint) | **Groups** | | | | |
| --- | --- | --- | --- | --- | --- |
| Sampling time: **21 days post-vaccination** | **Blood parameter (Monocytes, 10^3/µl)** | | | | |
| Replication | BS | BF | MS | MA | Unvaccinated |
| R1 (fish-1) | 0.37 | 0.54 | 0.51 | 0.53 | 0.44 |
| R1 (fish-2) | 0.37 | 0.47 | 0.46 | 0.46 | 0.42 |
| R1 (fish-3) | 0.34 | 0.46 | 0.47 | 0.48 | 0.43 |
| Average | 0.36 | 0.49 | 0.48 | 0.49 | 0.43 |
|  |  |  |  |  |  |
| R2 (fish-1) | 0.45 | 0.28 | 0.53 | 0.52 | 0.43 |
| R2 (fish-2) | 0.44 | 0.3 | 0.44 | 0.52 | 0.39 |
| R2 (fish-3) | 0.43 | 0.32 | 0.47 | 0.49 | 0.41 |
| Average | 0.44 | 0.3 | 0.48 | 0.51 | 0.41 |
|  |  |  |  |  |  |
| R3 (fish-1) | 0.29 | 0.42 | 0.42 | 0.42 | 0.47 |
| R3 (fish-2) | 0.32 | 0.35 | 0.42 | 0.51 | 0.44 |
| R3 (fish-3) | 0.35 | 0.37 | 0.42 | 0.45 | 0.41 |
| Average | 0.32 | 0.38 | 0.42 | 0.46 | 0.44 |

| 2. Blood was collected from 3 fish per replication (9 fish/group/timepoint) | **Groups** | | | | |
| --- | --- | --- | --- | --- | --- |
| Sampling time: **21 days post-vaccination** | **Blood parameter (Granulocytes, 10^3/µl)** | | | | |
| Replication | BS | BF | MS | MA | Unvaccinated |
| R1 (fish-1) | 4.74 | 7.34 | 7.45 | 4.17 | 6.45 |
| R1 (fish-2) | 5.78 | 6.33 | 6.75 | 4.13 | 5.41 |
| R1 (fish-3) | 3.94 | 5.47 | 7.94 | 4.21 | 6.44 |
| Average | 4.82 | 6.38 | 7.38 | 4.17 | 6.1 |
|  |  |  |  |  |  |
| R2 (fish-1) | 4.31 | 7.45 | 8.37 | 8.37 | 5.54 |
| R2 (fish-2) | 3.53 | 5.32 | 8.12 | 8.12 | 6.24 |
| R2 (fish-3) | 3.95 | 6.34 | 5.32 | 5.32 | 4.12 |
| Average | 3.93 | 6.37 | 7.27 | 7.27 | 5.3 |
|  |  |  |  |  |  |
| R3 (fish-1) | 5.12 | 5.62 | 4.38 | 7.44 | 6.45 |
| R3 (fish-2) | 3.11 | 3.67 | 2.91 | 7.42 | 5.54 |
| R3 (fish-3) | 4.13 | 3.82 | 3.66 | 5.93 | 5.35 |
| Average | 4.12 | 4.37 | 3.65 | 6.93 | 5.78 |

| 2. Blood was collected from 3 fish per replication (9 fish/group/timepoint) | **Groups** | | | | |
| --- | --- | --- | --- | --- | --- |
| Sampling time: **21 days post-vaccination** | **Blood parameter (Haemoglobin, g/dl)** | | | | |
| Replication | BS | BF | MS | MA | Unvaccinated |
| R1 (fish-1) | 4.14 | 6.11 | 4.85 | 5.56 | 7.45 |
| R1 (fish-2) | 3.42 | 7.11 | 5.81 | 6.24 | 6.41 |
| R1 (fish-3) | 5.55 | 5.32 | 3.95 | 4.34 | 5.28 |
| Average | 4.37 | 6.18 | 4.87 | 5.38 | 6.38 |
|  |  |  |  |  |  |
| R2 (fish-1) | 7.84 | 3.36 | 7.56 | 10.45 | 8.46 |
| R2 (fish-2) | 7.23 | 5.47 | 7.53 | 9.24 | 6.23 |
| R2 (fish-3) | 5.75 | 4.34 | 4.95 | 8.45 | 7.45 |
| Average | 6.94 | 4.39 | 6.68 | 9.38 | 7.38 |
|  |  |  |  |  |  |
| R3 (fish-1) | 4.34 | 5.34 | 7.83 | 7.56 | 6.53 |
| R3 (fish-2) | 3.46 | 3.45 | 8.89 | 4.25 | 5.12 |
| R3 (fish-3) | 2.34 | 4.35 | 6.95 | 4.33 | 4.22 |
| Average | 3.38 | 4.38 | 7.89 | 5.38 | 5.29 |

| 2. Blood was collected from 3 fish per replication (9 fish/group/timepoint) | **Groups** | | | | |
| --- | --- | --- | --- | --- | --- |
| Sampling time: **21 days post-vaccination** | **Blood parameter (MCH, pg)** | | | | |
| Replication | BS | BF | MS | MA | Unvaccinated |
| R1 (fish-1) | 18.34 | 13.13 | 20.45 | 19.12 | 13.16 |
| R1 (fish-2) | 17.24 | 12.37 | 18.53 | 21.12 | 11.34 |
| R1 (fish-3) | 16.56 | 8.34 | 16.73 | 17.63 | 15.34 |
| Average | 17.38 | 11.28 | 18.57 | 19.29 | 13.28 |
|  |  |  |  |  |  |
| R2 (fish-1) | 18.34 | 15.35 | 19.49 | 17.34 | 17.54 |
| R2 (fish-2) | 16.43 | 17.37 | 17.65 | 16.23 | 14.78 |
| R2 (fish-3) | 20.34 | 19.15 | 18.87 | 15.12 | 13.55 |
| Average | 18.37 | 17.29 | 18.67 | 16.23 | 15.29 |
|  |  |  |  |  |  |
| R3 (fish-1) | 14.38 | 18.45 | 13.45 | 16.83 | 11.55 |
| R3 (fish-2) | 17.67 | 15.35 | 12.27 | 13.95 | 14.52 |
| R3 (fish-3) | 16.76 | 15.34 | 11.45 | 12.36 | 11.34 |
| Average | 16.27 | 16.38 | 12.39 | 14.38 | 12.47 |

| 2. Blood was collected from 3 fish per replication (9 fish/group/timepoint) | **Groups** | | | | |
| --- | --- | --- | --- | --- | --- |
| Sampling time: **21 days post-vaccination** | **Blood parameter (MCHC, g/dl)** | | | | |
| Replication | BS | BF | MS | MA | Unvaccinated |
| R1 (fish-1) | 40.34 | 38.29 | 43.94 | 43.31 | 39.05 |
| R1 (fish-2) | 34.16 | 40.13 | 42.56 | 40.12 | 40.35 |
| R1 (fish-3) | 37.34 | 36.45 | 43.34 | 43.23 | 38.35 |
| Average | 37.28 | 38.29 | 43.28 | 42.22 | 39.25 |
|  |  |  |  |  |  |
| R2 (fish-1) | 45.14 | 43.23 | 38.27 | 50.04 | 46.15 |
| R2 (fish-2) | 40.25 | 41.29 | 36.05 | 51.26 | 46.32 |
| R2 (fish-3) | 41.15 | 39.35 | 37.19 | 47.14 | 43.34 |
| Average | 42.18 | 41.29 | 37.17 | 49.48 | 45.27 |
|  |  |  |  |  |  |
| R3 (fish-1) | 38.23 | 39.44 | 42.34 | 40.32 | 39.24 |
| R3 (fish-2) | 34.17 | 38.04 | 44.36 | 42.04 | 39.82 |
| R3 (fish-3) | 36.47 | 37.33 | 40.14 | 38.42 | 35.63 |
| Average | 36.29 | 38.27 | 42.28 | 40.26 | 38.23 |

| 2. Blood was collected from 3 fish per replication (9 fish/group/timepoint) | **Groups** | | | | |
| --- | --- | --- | --- | --- | --- |
| Sampling time: **21 days post-vaccination** | **Blood parameter (Haematocrit%)** | | | | |
| Replication | BS | BF | MS | MA | Unvaccinated |
| R1 (fish-1) | 22.14 | 19.34 | 20.24 | 24.34 | 26.34 |
| R1 (fish-2) | 19.63 | 20.36 | 21.98 | 21.84 | 25.34 |
| R1 (fish-3) | 19.34 | 18.14 | 17.45 | 23.84 | 24.34 |
| Average | 20.37 | 19.28 | 19.89 | 23.34 | 25.34 |
|  |  |  |  |  |  |
| R2 (fish-1) | 17.34 | 16.32 | 25.14 | 16.23 | 16.34 |
| R2 (fish-2) | 16.98 | 14.32 | 28.18 | 17.64 | 14.92 |
| R2 (fish-3) | 15.36 | 12.14 | 25.13 | 13.35 | 15.66 |
| Average | 16.56 | 14.26 | 26.15 | 15.74 | 15.64 |
|  |  |  |  |  |  |
| R3 (fish-1) | 16.43 | 23.42 | 14.32 | 19.23 | 12.33 |
| R3 (fish-2) | 19.52 | 20.13 | 15.55 | 20.45 | 14.93 |
| R3 (fish-3) | 13.34 | 23.02 | 13.54 | 18.07 | 10.42 |
| Average | 16.43 | 22.19 | 14.47 | 19.25 | 12.56 |

| 2. Blood was collected from 3 fish per replication (9 fish/group/timepoint) | **Groups** | | | | |
| --- | --- | --- | --- | --- | --- |
| Sampling time: **21 days post-vaccination** | **Immune parameter (Lysozyme, unit/ml)** | | | | |
| Replication | BS | BF | MS | MA | Unvaccinated |
| R1 (fish-1) | 29.34 | 25.34 | 32.95 | 27.53 | 29.34 |
| R1 (fish-2) | 27.26 | 26.23 | 35.31 | 29.84 | 26.62 |
| R1 (fish-3) | 26.14 | 23.34 | 36.32 | 25.34 | 23.45 |
| Average | 27.58 | 24.97 | 34.86 | 27.57 | 26.47 |
|  |  |  |  |  |  |
| R2 (fish-1) | 24.29 | 26.94 | 39.76 | 39.45 | 34.44 |
| R2 (fish-2) | 21.74 | 23.36 | 38.19 | 40.23 | 30.76 |
| R2 (fish-3) | 21.41 | 26.65 | 35.87 | 33.24 | 33.35 |
| Average | 22.48 | 25.65 | 37.94 | 37.64 | 32.85 |
|  |  |  |  |  |  |
| R3 (fish-1) | 18.34 | 34.24 | 32.34 | 34.52 | 29.34 |
| R3 (fish-2) | 20.13 | 35.14 | 33.32 | 29.22 | 32.14 |
| R3 (fish-3) | 17.24 | 31.24 | 28.45 | 30.34 | 28.34 |
| Average | 18.57 | 33.54 | 31.37 | 31.36 | 29.94 |

| 2. Blood was collected from 3 fish per replication (9 fish/group/timepoint) | **Groups** | | | | |
| --- | --- | --- | --- | --- | --- |
| Sampling time: **21 days post-vaccination** | **Immune parameter (Phagocytosis%)** | | | | |
| Replication | BS | BF | MS | MA | Unvaccinated |
| R1 (fish-1) | 26.34 | 34.56 | 36.36 | 39.45 | 39.43 |
| R1 (fish-2) | 25.22 | 35.57 | 38.28 | 33.65 | 33.39 |
| R1 (fish-3) | 23.05 | 31.39 | 31.44 | 36.31 | 33.56 |
| Average | 24.87 | 33.84 | 35.36 | 36.47 | 35.46 |
|  |  |  |  |  |  |
| R2 (fish-1) | 23.04 | 39.45 | 38.45 | 49.34 | 42.34 |
| R2 (fish-2) | 21.09 | 37.34 | 35.69 | 48.05 | 40.29 |
| R2 (fish-3) | 17.04 | 35.29 | 35.27 | 44.06 | 40.22 |
| Average | 20.39 | 37.36 | 36.47 | 47.15 | 40.95 |
|  |  |  |  |  |  |
| R3 (fish-1) | 29.44 | 35.17 | 44.34 | 38.45 | 34.23 |
| R3 (fish-2) | 26.93 | 32.16 | 44.36 | 36.43 | 34.02 |
| R3 (fish-3) | 25.74 | 30.05 | 40.24 | 31.23 | 29.04 |
| Average | 27.37 | 32.46 | 42.98 | 35.37 | 32.43 |

| 2. Blood was collected from 3 fish per replication (9 fish/group/timepoint) | **Groups** | | | | |
| --- | --- | --- | --- | --- | --- |
| Sampling time: **21 days post-vaccination** | **Immune parameter (Antibody level)** | | | | |
| Replication | BS | BF | MS | MA | Unvaccinated |
| R1 (fish-1) | 0.28 | 0.52 | 0.75 | 0.68 | 0.64 |
| R1 (fish-2) | 0.16 | 0.36 | 0.42 | 0.75 | 0.29 |
| R1 (fish-3) | 0.22 | 0.41 | 0.75 | 0.28 | 0.51 |
| Average | 0.22 | 0.43 | 0.64 | 0.57 | 0.48 |
|  |  |  |  |  |  |
| R2 (fish-1) | 0.25 | 0.43 | 0.78 | 0.33 | 0.51 |
| R2 (fish-2) | 0.32 | 0.27 | 0.66 | 0.52 | 0.58 |
| R2 (fish-3) | 0.21 | 0.23 | 0.45 | 0.35 | 0.41 |
| Average | 0.26 | 0.31 | 0.63 | 0.4 | 0.5 |
|  |  |  |  |  |  |
| R3 (fish-1) | 0.32 | 0.56 | 0.64 | 0.37 | 0.66 |
| R3 (fish-2) | 0.23 | 0.6 | 0.75 | 0.68 | 0.42 |
| R3 (fish-3) | 0.26 | 0.25 | 0.41 | 0.45 | 0.51 |
| Average | 0.27 | 0.47 | 0.6 | 0.5 | 0.53 |

**Note:** Bivalent Spray (BS); Bivalent Formulate (BF); Monovalent *S. iniae* (MS); Monovalent *A. hydrophila* (MA). Data were presented in main manuscript **Tables 1 and 2.**

| 3. Blood was collected from 3 fish per replication (9 fish/group/timepoint) | **Groups** | | | | |
| --- | --- | --- | --- | --- | --- |
| Sampling time: **49 days post-vaccination** | **Blood parameter (Erythrocytes, 10^6/µl)** | | | | |
| Replication | BS | BF | MS | MA | Unvaccinated |
| R1 (fish-1) | 5.65 | 6.48 | 9.46 | 4.56 | 4.48 |
| R1 (fish-2) | 3.72 | 7.27 | 8.98 | 6.81 | 3.54 |
| R1 (fish-3) | 3.47 | 5.24 | 6.85 | 5.49 | 4.73 |
| Average | 4.28 | 6.33 | 8.43 | 5.62 | 4.25 |
|  |  |  |  |  |  |
| R2 (fish-1) | 2.27 | 3.46 | 8.58 | 8.44 | 6.48 |
| R2 (fish-2) | 5.21 | 4.22 | 5.53 | 6.31 | 6.72 |
| R2 (fish-3) | 2.27 | 1.95 | 6.47 | 6.73 | 4.68 |
| Average | 3.25 | 3.21 | 6.86 | 7.16 | 5.96 |
|  |  |  |  |  |  |
| R3 (fish-1) | 6.45 | 5.28 | 5.37 | 9.85 | 8.94 |
| R3 (fish-2) | 4.35 | 4.33 | 4.17 | 7.54 | 9.64 |
| R3 (fish-3) | 4.56 | 3.56 | 3.27 | 8.53 | 7.94 |
| Average | 5.12 | 4.39 | 4.27 | 8.64 | 8.84 |

**Sampling time:** **49 days post-vaccination**

| 3. Blood was collected from 3 fish per replication (9 fish/group/timepoint) | **Groups** | | | | |
| --- | --- | --- | --- | --- | --- |
| Sampling time: **49 days post-vaccination** | **Blood parameter (Thrombocytes, 10^3/µl)** | | | | |
| Replication | BS | BF | MS | MA | Unvaccinated |
| R1 (fish-1) | 15.45 | 18.59 | 26.52 | 20.34 | 19.67 |
| R1 (fish-2) | 12.34 | 18.33 | 25.73 | 19.53 | 22.54 |
| R1 (fish-3) | 12.59 | 16.24 | 24.94 | 18.84 | 18.84 |
| Average | 13.46 | 17.72 | 25.73 | 19.57 | 20.35 |
|  |  |  |  |  |  |
| R2 (fish-1) | 17.73 | 27.24 | 34.19 | 23.45 | 19.34 |
| R2 (fish-2) | 16.28 | 28.12 | 31.18 | 23.65 | 17.63 |
| R2 (fish-3) | 15.43 | 29.27 | 31.14 | 22.44 | 18.41 |
| Average | 16.48 | 28.21 | 32.17 | 23.18 | 18.46 |
|  |  |  |  |  |  |
| R3 (fish-1) | 24.37 | 23.43 | 23.32 | 32.34 | 25.78 |
| R3 (fish-2) | 23.48 | 25.83 | 20.49 | 31.53 | 23.46 |
| R3 (fish-3) | 22.56 | 25.56 | 21.44 | 31.32 | 24.35 |
| Average | 23.47 | 24.94 | 21.75 | 31.73 | 24.53 |

| 3. Blood was collected from 3 fish per replication (9 fish/group/timepoint) | **Groups** | | | | |
| --- | --- | --- | --- | --- | --- |
| Sampling time: **49 days post-vaccination** | **Blood parameter (Leucocytes, 10^3/µl)** | | | | |
| Replication | BS | BF | MS | MA | Unvaccinated |
| R1 (fish-1) | 28.39 | 39.56 | 41.23 | 52.45 | 38.75 |
| R1 (fish-2) | 26.66 | 38.44 | 42.13 | 50.55 | 39.75 |
| R1 (fish-3) | 27.63 | 37.74 | 43.42 | 48.74 | 37.75 |
| Average | 27.56 | 38.58 | 42.26 | 50.58 | 38.75 |
|  |  |  |  |  |  |
| R2 (fish-1) | 27.15 | 40.12 | 42.34 | 44.34 | 45.44 |
| R2 (fish-2) | 25.23 | 38.19 | 42.37 | 42.54 | 43.52 |
| R2 (fish-3) | 27.24 | 39.23 | 40.24 | 45.54 | 43.55 |
| Average | 26.54 | 39.18 | 41.65 | 44.14 | 44.17 |
|  |  |  |  |  |  |
| R3 (fish-1) | 30.45 | 42.24 | 47.26 | 49.16 | 45.34 |
| R3 (fish-2) | 28.34 | 40.35 | 45.35 | 47.44 | 43.28 |
| R3 (fish-3) | 30.16 | 38.25 | 44.25 | 46.32 | 42.33 |
| Average | 29.65 | 40.28 | 45.62 | 47.64 | 43.65 |

| 3. Blood was collected from 3 fish per replication (9 fish/group/timepoint) | **Groups** | | | | |
| --- | --- | --- | --- | --- | --- |
| Sampling time: **49 days post-vaccination** | **Blood parameter (Lymphocytes, 10^3/µl)** | | | | |
| Replication | BS | BF | MS | MA | Unvaccinated |
| R1 (fish-1) | 20.43 | 26.34 | 29.23 | 31.35 | 30.23 |
| R1 (fish-2) | 20.34 | 25.73 | 27.69 | 31.28 | 29.61 |
| R1 (fish-3) | 18.24 | 24.34 | 29.75 | 28.15 | 28.54 |
| Average | 19.67 | 25.47 | 28.89 | 30.26 | 29.46 |
|  |  |  |  |  |  |
| R2 (fish-1) | 16.34 | 22.34 | 29.34 | 26.45 | 26.42 |
| R2 (fish-2) | 16.59 | 23.36 | 27.82 | 27.65 | 25.23 |
| R2 (fish-3) | 13.45 | 25.34 | 28.55 | 26.57 | 24.16 |
| Average | 15.46 | 23.68 | 28.57 | 26.89 | 25.27 |
|  |  |  |  |  |  |
| R3 (fish-1) | 21.34 | 21.83 | 25.14 | 29.25 | 25.56 |
| R3 (fish-2) | 21.81 | 20.54 | 25.17 | 29.25 | 26.67 |
| R3 (fish-3) | 19.43 | 22.34 | 26.13 | 29.25 | 27.45 |
| Average | 20.86 | 21.57 | 25.48 | 29.25 | 26.56 |

| 3. Blood was collected from 3 fish per replication (9 fish/group/timepoint) | **Groups** | | | | |
| --- | --- | --- | --- | --- | --- |
| Sampling time: **49 days post-vaccination** | **Blood parameter (Monocytes, 10^3/µl)** | | | | |
| Replication | BS | BF | MS | MA | Unvaccinated |
| R1 (fish-1) | 0.39 | 0.53 | 0.56 | 0.56 | 0.55 |
| R1 (fish-2) | 0.43 | 0.42 | 0.69 | 0.52 | 0.49 |
| R1 (fish-3) | 0.32 | 0.52 | 0.49 | 0.42 | 0.46 |
| Average | 0.38 | 0.49 | 0.58 | 0.5 | 0.5 |
|  |  |  |  |  |  |
| R2 (fish-1) | 0.41 | 0.54 | 0.64 | 0.56 | 0.54 |
| R2 (fish-2) | 0.44 | 0.53 | 0.52 | 0.67 | 0.65 |
| R2 (fish-3) | 0.38 | 0.46 | 0.52 | 0.63 | 0.46 |
| Average | 0.41 | 0.51 | 0.56 | 0.62 | 0.55 |
|  |  |  |  |  |  |
| R3 (fish-1) | 0.32 | 0.54 | 0.46 | 0.56 | 0.54 |
| R3 (fish-2) | 0.42 | 0.46 | 0.35 | 0.54 | 0.62 |
| R3 (fish-3) | 0.37 | 0.44 | 0.42 | 0.46 | 0.43 |
| Average | 0.37 | 0.48 | 0.41 | 0.52 | 0.53 |

| 3. Blood was collected from 3 fish per replication (9 fish/group/timepoint) | **Groups** | | | | |
| --- | --- | --- | --- | --- | --- |
| Sampling time: **49 days post-vaccination** | **Blood parameter (Granulocytes, 10^3/µl)** | | | | |
| Replication | BS | BF | MS | MA | Unvaccinated |
| R1 (fish-1) | 5.34 | 6.26 | 9.67 | 6.45 | 7.34 |
| R1 (fish-2) | 3.36 | 4.46 | 8.94 | 6.52 | 7.25 |
| R1 (fish-3) | 4.44 | 5.45 | 7.73 | 7.22 | 5.12 |
| Average | 4.38 | 5.39 | 8.78 | 6.73 | 6.57 |
|  |  |  |  |  |  |
| R2 (fish-1) | 4.31 | 6.23 | 8.15 | 5.34 | 5.45 |
| R2 (fish-2) | 5.11 | 6.45 | 9.08 | 5.38 | 5.94 |
| R2 (fish-3) | 3.24 | 5.23 | 7.13 | 5.45 | 4.78 |
| Average | 4.22 | 5.97 | 8.12 | 5.39 | 5.39 |
|  |  |  |  |  |  |
| R3 (fish-1) | 4.15 | 6.45 | 8.27 | 6.95 | 7.42 |
| R3 (fish-2) | 3.19 | 4.68 | 6.49 | 5.65 | 6.35 |
| R3 (fish-3) | 2.47 | 6.45 | 7.32 | 6.51 | 5.34 |
| Average | 3.27 | 5.86 | 7.36 | 6.37 | 6.37 |

| 3. Blood was collected from 3 fish per replication (9 fish/group/timepoint) | **Groups** | | | | |
| --- | --- | --- | --- | --- | --- |
| Sampling time: **49 days post-vaccination** | **Blood parameter (Haemoglobin, g/dl)** | | | | |
| Replication | BS | BF | MS | MA | Unvaccinated |
| R1 (fish-1) | 5.44 | 4.34 | 5.34 | 6.45 | 7.23 |
| R1 (fish-2) | 5.67 | 3.62 | 5.26 | 7.95 | 5.27 |
| R1 (fish-3) | 3.56 | 2.75 | 3.41 | 6.54 | 7.45 |
| Average | 4.89 | 3.57 | 4.67 | 6.98 | 6.65 |
|  |  |  |  |  |  |
| R2 (fish-1) | 6.45 | 9.89 | 10.67 | 5.34 | 4.44 |
| R2 (fish-2) | 5.74 | 6.86 | 9.34 | 3.59 | 3.78 |
| R2 (fish-3) | 5.36 | 6.89 | 6.78 | 4.18 | 4.62 |
| Average | 5.85 | 7.88 | 8.93 | 4.37 | 4.28 |
|  |  |  |  |  |  |
| R3 (fish-1) | 5.12 | 6.43 | 6.62 | 10.35 | 8.34 |
| R3 (fish-2) | 5.17 | 5.31 | 7.85 | 9.94 | 9.46 |
| R3 (fish-3) | 3.45 | 5.45 | 5.87 | 8.75 | 8.57 |
| Average | 4.58 | 5.73 | 6.78 | 9.68 | 8.79 |

| 3. Blood was collected from 3 fish per replication (9 fish/group/timepoint) | Groups | | | | |
| --- | --- | --- | --- | --- | --- |
| Sampling time: **49 days post-vaccination** | **Blood parameter (MCH, pg)** | | | | |
| Replication | BS | BF | MS | MA | Unvaccinated |
| R1 (fish-1) | 21.53 | 20.23 | 13.13 | 14.15 | 14.34 |
| R1 (fish-2) | 19.74 | 23.42 | 13.29 | 13.95 | 12.33 |
| R1 (fish-3) | 20.44 | 21.15 | 12.22 | 12.34 | 11.31 |
| Average | 20.57 | 21.6 | 12.88 | 13.48 | 12.66 |
|  |  |  |  |  |  |
| R2 (fish-1) | 16.74 | 15.34 | 15.49 | 23.36 | 21.34 |
| R2 (fish-2) | 14.81 | 13.66 | 14.75 | 21.94 | 19.54 |
| R2 (fish-3) | 14.53 | 12.34 | 12.48 | 20.64 | 20.47 |
| Average | 15.36 | 13.78 | 14.24 | 21.98 | 20.45 |
|  |  |  |  |  |  |
| R3 (fish-1) | 16.41 | 17.33 | 22.22 | 16.45 | 14.45 |
| R3 (fish-2) | 15.54 | 14.23 | 19.46 | 16.29 | 15.68 |
| R3 (fish-3) | 14.16 | 15.03 | 20.33 | 13.97 | 13.85 |
| Average | 15.37 | 15.53 | 20.67 | 15.57 | 14.66 |

| 3. Blood was collected from 3 fish per replication (9 fish/group/timepoint) | **Groups** | | | | |
| --- | --- | --- | --- | --- | --- |
| Sampling time**: 49 days post-vaccination** | **Blood parameter (MCHC, g/dl)** | | | | |
| Replication | BS | BF | MS | MA | Unvaccinated |
| R1 (fish-1) | 40.32 | 36.65 | 46.15 | 48.51 | 41.34 |
| R1 (fish-2) | 36.73 | 33.98 | 43.85 | 46.64 | 43.25 |
| R1 (fish-3) | 38.66 | 36.68 | 45.78 | 45.34 | 42.22 |
| Average | 38.57 | 35.77 | 45.26 | 46.83 | 42.27 |
|  |  |  |  |  |  |
| R2 (fish-1) | 35.45 | 41.94 | 36.82 | 37.34 | 39.15 |
| R2 (fish-2) | 32.16 | 40.65 | 36.53 | 41.91 | 36.48 |
| R2 (fish-3) | 35.17 | 39.45 | 36.81 | 40.42 | 39.24 |
| Average | 34.26 | 40.68 | 36.72 | 39.89 | 38.29 |
|  |  |  |  |  |  |
| R3 (fish-1) | 36.47 | 39.51 | 41.23 | 34.15 | 50.79 |
| R3 (fish-2) | 33.89 | 39.23 | 39.36 | 32.55 | 48.81 |
| R3 (fish-3) | 37.58 | 37.45 | 40.25 | 34.34 | 49.74 |
| Average | 35.98 | 38.73 | 40.28 | 33.68 | 49.78 |

| 3. Blood was collected from 3 fish per replication (9 fish/group/timepoint) | **Groups** | | | | |
| --- | --- | --- | --- | --- | --- |
| Sampling time: **49 days post-vaccination** | **Blood parameter (Haematocrit%)** | | | | |
| Replication | BS | BF | MS | MA | Unvaccinated |
| R1 (fish-1) | 15.34 | 21.23 | 22.45 | 15.34 | 17.36 |
| R1 (fish-2) | 14.36 | 20.93 | 19.94 | 15.29 | 21.37 |
| R1 (fish-3) | 13.35 | 20.36 | 23.55 | 14.25 | 19.35 |
| Average | 14.35 | 20.84 | 21.98 | 14.96 | 19.36 |
|  |  |  |  |  |  |
| R2 (fish-1) | 21.31 | 14.34 | 17.34 | 24.46 | 17.33 |
| R2 (fish-2) | 23.23 | 13.69 | 17.33 | 23.31 | 14.84 |
| R2 (fish-3) | 20.14 | 15.35 | 15.64 | 23.15 | 14.45 |
| Average | 21.56 | 14.46 | 16.77 | 23.64 | 15.54 |
|  |  |  |  |  |  |
| R3 (fish-1) | 14.03 | 16.32 | 17.34 | 22.23 | 19.33 |
| R3 (fish-2) | 12.45 | 13.42 | 19.35 | 19.83 | 17.48 |
| R3 (fish-3) | 14.23 | 14.51 | 16.83 | 19.41 | 19.23 |
| Average | 13.57 | 14.75 | 17.84 | 20.49 | 18.68 |

| 3. Blood was collected from 3 fish per replication (9 fish/group/timepoint) | **Groups** | | | | |
| --- | --- | --- | --- | --- | --- |
| Sampling time: **49 days post-vaccination** | **Immune parameter (Lysozyme, unit/ml)** | | | | |
| Replication | BS | BF | MS | MA | Unvaccinated |
| R1 (fish-1) | 21.33 | 32.34 | 39.44 | 44.33 | 38.34 |
| R1 (fish-2) | 23.41 | 31.71 | 37.33 | 42.31 | 35.78 |
| R1 (fish-3) | 25.61 | 33.21 | 38.34 | 44.37 | 34.39 |
| Average | 23.45 | 32.42 | 38.37 | 43.67 | 36.17 |
|  |  |  |  |  |  |
| R2 (fish-1) | 26.03 | 31.18 | 49.33 | 30.21 | 43.45 |
| R2 (fish-2) | 22.16 | 31.28 | 47.51 | 32.11 | 45.82 |
| R2 (fish-3) | 24.32 | 29.13 | 42.87 | 34.43 | 40.51 |
| Average | 24.17 | 30.53 | 46.57 | 32.25 | 43.26 |
|  |  |  |  |  |  |
| R3 (fish-1) | 27.34 | 38.63 | 39.23 | 35.34 | 36.34 |
| R3 (fish-2) | 24.19 | 35.64 | 38.94 | 37.19 | 34.84 |
| R3 (fish-3) | 25.15 | 35.62 | 37.81 | 37.36 | 35.11 |
| Average | 25.56 | 36.63 | 38.66 | 36.63 | 35.43 |

| 3. Blood was collected from 3 fish per replication (9 fish/group/timepoint) | **Groups** | | | | |
| --- | --- | --- | --- | --- | --- |
| Sampling time**: 49 days post-vaccination** | **Immunie parameter (Phagocytosis%)** | | | | |
| Replication | BS | BF | MS | MA | Unvaccinated |
| R1 (fish-1) | 26.43 | 29.14 | 39.65 | 35.34 | 41.06 |
| R1 (fish-2) | 27.25 | 29.12 | 34.72 | 33.59 | 40.24 |
| R1 (fish-3) | 25.49 | 27.12 | 35.85 | 33.55 | 39.12 |
| Average | 26.39 | 28.46 | 36.74 | 34.16 | 40.14 |
|  |  |  |  |  |  |
| R2 (fish-1) | 17.34 | 40.45 | 42.34 | 49.56 | 45.74 |
| R2 (fish-2) | 19.84 | 37.76 | 38.42 | 47.64 | 46.65 |
| R2 (fish-3) | 20.33 | 37.56 | 37.53 | 48.54 | 43.96 |
| Average | 19.17 | 38.59 | 39.43 | 48.58 | 45.45 |
|  |  |  |  |  |  |
| R3 (fish-1) | 26.53 | 32.36 | 46.45 | 37.14 | 32.23 |
| R3 (fish-2) | 23.42 | 32.53 | 47.59 | 35.18 | 30.85 |
| R3 (fish-3) | 26.34 | 32.52 | 45.34 | 36.16 | 30.43 |
| Average | 25.43 | 32.47 | 46.46 | 36.16 | 31.17 |

| 3. Blood was collected from 3 fish per replication (9 fish/group/timepoint) | **Groups** | | | | |
| --- | --- | --- | --- | --- | --- |
| Sampling time: **49 days post-vaccination** | **Immune parameter (Antibody level)** | | | | |
| Replication | BS | BF | MS | MA | Unvaccinated |
| R1 (fish-1) | 0.13 | 0.57 | 0.85 | 0.76 | 0.78 |
| R1 (fish-2) | 0.16 | 0.52 | 0.76 | 0.62 | 0.77 |
| R1 (fish-3) | 0.19 | 0.62 | 0.73 | 0.54 | 0.64 |
| Average | 0.16 | 0.57 | 0.78 | 0.64 | 0.73 |
|  |  |  |  |  |  |
| R2 (fish-1) | 0.22 | 0.46 | 0.73 | 0.77 | 0.87 |
| R2 (fish-2) | 0.21 | 0.56 | 0.79 | 0.71 | 0.86 |
| R2 (fish-3) | 0.26 | 0.66 | 0.73 | 0.65 | 0.64 |
| Average | 0.23 | 0.56 | 0.75 | 0.71 | 0.79 |
|  |  |  |  |  |  |
| R3 (fish-1) | 0.26 | 0.64 | 0.74 | 0.91 | 0.63 |
| R3 (fish-2) | 0.27 | 0.57 | 0.89 | 0.77 | 0.56 |
| R3 (fish-3) | 0.19 | 0.65 | 0.77 | 0.87 | 0.61 |
| Average | 0.24 | 0.62 | 0.8 | 0.85 | 0.6 |

**Note:** Bivalent Spray (BS); Bivalent Formulate (BF); Monovalent *S. iniae* (MS); Monovalent *A. hydrophila* (MA). Data were presented in main manuscript **Tables 1 and 2.**

| 4. Blood was collected from 3 fish per replication (9 fish/group/timepoint) | **Groups** | | | |
| --- | --- | --- | --- | --- |
| Sampling time: **96 hours post-infection with *S. iniae*** | **Blood parameter (Erythrocytes, 10^6/µl)** | | | |
| Replication | BS | BF | MS | Unvaccinated |
| R1 (fish-1) | 2.36 | 5.44 | 5.73 | 5.95 |
| R1 (fish-2) | 2.86 | 4.42 | 6.67 | 6.67 |
| R1 (fish-3) | 2.82 | 6.25 | 5.48 | 7.63 |
| Average | 2.68 | 5.37 | 5.96 | 6.75 |
|  |  |  |  |  |
| R2 (fish-1) | 3.23 | 5.56 | 8.49 | 9.43 |
| R2 (fish-2) | 3.18 | 4.85 | 8.97 | 8.66 |
| R2 (fish-3) | 2.56 | 6.48 | 6.36 | 8.76 |
| Average | 2.99 | 5.63 | 7.94 | 8.95 |
|  |  |  |  |  |
| R3 (fish-1) | 3.13 | 5.48 | 6.47 | 5.68 |
| R3 (fish-2) | 3.78 | 3.59 | 7.36 | 4.59 |
| R3 (fish-3) | 2.87 | 4.67 | 6.78 | 5.84 |
| Average | 3.26 | 4.58 | 6.87 | 5.37 |

**Sampling time:** **96 hours post-infection with *S. iniae***

| 4. Blood was collected from 3 fish per replication (9 fish/group/timepoint) | **Groups** | | | |
| --- | --- | --- | --- | --- |
| Sampling time: **96 hours post-infection with *S. iniae*** | **Blood parameter (Thrombocytes, 10^3/µl)** | | | |
| Replication | BS | BF | MS | Unvaccinated |
| R1 (fish-1) | 14.33 | 14.56 | 20.32 | 29.43 |
| R1 (fish-2) | 13.58 | 13.63 | 19.44 | 28.39 |
| R1 (fish-3) | 13.43 | 15.55 | 20.12 | 27.26 |
| Average | 13.78 | 14.58 | 19.96 | 28.36 |
|  |  |  |  |  |
| R2 (fish-1) | 21.66 | 27.45 | 23.45 | 23.41 |
| R2 (fish-2) | 21.61 | 24.58 | 20.45 | 25.33 |
| R2 (fish-3) | 18.74 | 28.34 | 20.51 | 25.81 |
| Average | 20.67 | 26.79 | 21.47 | 24.85 |
|  |  |  |  |  |
| R3 (fish-1) | 14.34 | 19.54 | 28.44 | 21.45 |
| R3 (fish-2) | 12.62 | 15.84 | 27.66 | 19.94 |
| R3 (fish-3) | 12.55 | 20.63 | 24.48 | 20.68 |
| Average | 13.17 | 18.67 | 26.86 | 20.69 |

| 4. Blood was collected from 3 fish per replication (9 fish/group/timepoint) | Groups | | | |
| --- | --- | --- | --- | --- |
| Sampling time: **96 hours post-infection with *S. iniae*** | **Blood parameter (Leucocytes, 10^3/µl)** | | | |
| Replication | BS | BF | MS | Unvaccinated |
| R1 (fish-1) | 23.55 | 26.57 | 32.15 | 34.23 |
| R1 (fish-2) | 22.72 | 26.98 | 28.19 | 33.67 |
| R1 (fish-3) | 25.34 | 24.39 | 31.13 | 32.54 |
| Average | 23.87 | 25.98 | 30.49 | 33.48 |
|  |  |  |  |  |
| R2 (fish-1) | 14.34 | 22.12 | 34.72 | 29.26 |
| R2 (fish-2) | 17.22 | 20.35 | 32.71 | 30.27 |
| R2 (fish-3) | 15.39 | 25.45 | 33.76 | 28.28 |
| Average | 15.65 | 22.64 | 33.73 | 29.27 |
|  |  |  |  |  |
| R3 (fish-1) | 14.55 | 22.34 | 35.12 | 26.12 |
| R3 (fish-2) | 17.69 | 25.63 | 36.64 | 23.42 |
| R3 (fish-3) | 17.47 | 22.41 | 34.32 | 23.51 |
| Average | 16.57 | 23.46 | 35.36 | 24.35 |

| 4. Blood was collected from 3 fish per replication (9 fish/group/timepoint) | **Groups** | | | |
| --- | --- | --- | --- | --- |
| Sampling time: **96 hours post-infection with *S. iniae*** | **Blood parameter (Lymphocytes, 10^3/µl)** | | | |
| Replication | BS | BF | MS | Unvaccinated |
| R1 (fish-1) | 24.82 | 28.83 | 31.13 | 33.45 |
| R1 (fish-2) | 22.74 | 27.81 | 29.12 | 31.44 |
| R1 (fish-3) | 23.45 | 25.74 | 31.16 | 32.52 |
| Average | 23.67 | 27.46 | 30.47 | 32.47 |
|  |  |  |  |  |
| R2 (fish-1) | 23.21 | 29.12 | 31.34 | 29.29 |
| R2 (fish-2) | 22.13 | 28.71 | 28.47 | 27.26 |
| R2 (fish-3) | 25.34 | 28.39 | 29.23 | 32.49 |
| Average | 23.56 | 28.74 | 29.68 | 29.68 |
|  |  |  |  |  |
| R3 (fish-1) | 16.35 | 28.48 | 29.15 | 28.29 |
| R3 (fish-2) | 18.39 | 26.57 | 26.79 | 27.47 |
| R3 (fish-3) | 17.55 | 25.41 | 29.14 | 29.32 |
| Average | 17.43 | 26.82 | 28.36 | 28.36 |

| 4. Blood was collected from 3 fish per replication (9 fish/group/timepoint) | **Groups** | | | |
| --- | --- | --- | --- | --- |
| Sampling time**: 96 hours post-infection with *S. iniae*** | **Blood parameter (Monocytes, 10^3/µl)** | | | |
| Replication | BS | BF | MS | Unvaccinated |
| R1 (fish-1) | 0.22 | 0.32 | 0.32 | 0.32 |
| R1 (fish-2) | 0.26 | 0.29 | 0.38 | 0.38 |
| R1 (fish-3) | 0.24 | 0.41 | 0.32 | 0.29 |
| Average | 0.24 | 0.34 | 0.34 | 0.33 |
|  |  |  |  |  |
| R2 (fish-1) | 0.28 | 0.29 | 0.36 | 0.37 |
| R2 (fish-2) | 0.27 | 0.31 | 0.28 | 0.32 |
| R2 (fish-3) | 0.26 | 0.24 | 0.32 | 0.36 |
| Average | 0.27 | 0.28 | 0.32 | 0.35 |
|  |  |  |  |  |
| R3 (fish-1) | 0.17 | 0.24 | 0.39 | 0.29 |
| R3 (fish-2) | 0.24 | 0.37 | 0.34 | 0.33 |
| R3 (fish-3) | 0.19 | 0.23 | 0.32 | 0.28 |
| Average | 0.2 | 0.28 | 0.35 | 0.3 |

| 4. Blood was collected from 3 fish per replication (9 fish/group/timepoint) | **Groups** | | | |
| --- | --- | --- | --- | --- |
| Sampling time**: 96 hours post-infection with *S. iniae*** | **Blood parameter (Granulocytes, 10^3/µl)** | | | |
| Replication | BS | BF | MS | Unvaccinated |
| R1 (fish-1) | 3.21 | 6.34 | 6.14 | 5.67 |
| R1 (fish-2) | 3.55 | 4.46 | 4.76 | 6.23 |
| R1 (fish-3) | 4.13 | 5.31 | 5.24 | 5.38 |
| Average | 3.63 | 5.37 | 5.38 | 5.76 |
|  |  |  |  |  |
| R2 (fish-1) | 2.95 | 6.45 | 7.88 | 7.16 |
| R2 (fish-2) | 2.87 | 4.13 | 8.63 | 7.18 |
| R2 (fish-3) | 2.43 | 5.26 | 7.34 | 5.67 |
| Average | 2.75 | 5.28 | 7.95 | 6.67 |
|  |  |  |  |  |
| R3 (fish-1) | 4.13 | 4.26 | 5.34 | 7.45 |
| R3 (fish-2) | 2.67 | 3.19 | 5.42 | 5.41 |
| R3 (fish-3) | 4.12 | 5.33 | 5.38 | 4.36 |
| Average | 3.64 | 4.26 | 5.38 | 5.74 |

| 4. Blood was collected from 3 fish per replication (9 fish/group/timepoint) | **Groups** | | | |
| --- | --- | --- | --- | --- |
| Sampling time: **96 hours post-infection with *S. iniae*** | **Blood parameter (Haemoglobin, g/dl)** | | | |
| Replication | BS | BF | MS | Unvaccinated |
| R1 (fish-1) | 5.29 | 7.44 | 6.45 | 6.45 |
| R1 (fish-2) | 5.21 | 6.63 | 6.96 | 7.65 |
| R1 (fish-3) | 6.84 | 5.34 | 5.67 | 6.45 |
| Average | 5.78 | 6.47 | 6.36 | 6.85 |
|  |  |  |  |  |
| R2 (fish-1) | 1.93 | 5.68 | 6.45 | 8.57 |
| R2 (fish-2) | 2.56 | 5.35 | 6.35 | 8.26 |
| R2 (fish-3) | 3.22 | 6.25 | 7.45 | 6.69 |
| Average | 2.57 | 5.76 | 6.75 | 7.84 |
|  |  |  |  |  |
| R3 (fish-1) | 3.37 | 5.36 | 6.48 | 6.35 |
| R3 (fish-2) | 3.14 | 6.29 | 5.21 | 7.16 |
| R3 (fish-3) | 4.17 | 4.49 | 5.23 | 5.57 |
| Average | 3.56 | 5.38 | 5.64 | 6.36 |

| 4. Blood was collected from 3 fish per replication (9 fish/group/timepoint) | **Groups** | | | |
| --- | --- | --- | --- | --- |
| Sampling time: **96 hours post-infection with *S. iniae*** | **Blood parameter (MCH, pg)** | | | |
| Replication | BS | BF | MS | Unvaccinated |
| R1 (fish-1) | 14.78 | 16.45 | 11.23 | 18.13 |
| R1 (fish-2) | 17.82 | 15.57 | 9.48 | 16.52 |
| R1 (fish-3) | 17.56 | 14.66 | 11.24 | 17.34 |
| Average | 16.72 | 15.56 | 10.65 | 17.33 |
|  |  |  |  |  |
| R2 (fish-1) | 19.34 | 13.41 | 19.34 | 14.34 |
| R2 (fish-2) | 18.28 | 9.81 | 17.29 | 11.83 |
| R2 (fish-3) | 15.33 | 10.14 | 19.38 | 14.21 |
| Average | 17.65 | 11.12 | 18.67 | 13.46 |
|  |  |  |  |  |
| R3 (fish-1) | 13.51 | 13.53 | 8.34 | 12.34 |
| R3 (fish-2) | 10.83 | 12.35 | 9.54 | 13.23 |
| R3 (fish-3) | 12.74 | 11.83 | 10.29 | 15.14 |
| Average | 12.36 | 12.57 | 9.39 | 13.57 |

| 4. Blood was collected from 3 fish per replication (9 fish/group/timepoint) | **Groups** | | | |
| --- | --- | --- | --- | --- |
| Sampling time: **96 hours post-infection with *S. iniae*** | **Blood parameter (MCHC, g/dl)** | | | |
| Replication | BS | BF | MS | Unvaccinated |
| R1 (fish-1) | 21.41 | 29.44 | 21.26 | 21.44 |
| R1 (fish-2) | 23.75 | 25.57 | 20.39 | 25.71 |
| R1 (fish-3) | 25.52 | 27.34 | 20.03 | 25.63 |
| Average | 23.56 | 27.45 | 20.56 | 24.26 |
|  |  |  |  |  |
| R2 (fish-1) | 23.41 | 28.34 | 34.23 | 26.34 |
| R2 (fish-2) | 19.55 | 24.52 | 32.21 | 28.66 |
| R2 (fish-3) | 21.45 | 26.22 | 31.24 | 27.44 |
| Average | 21.47 | 26.36 | 32.56 | 27.48 |
|  |  |  |  |  |
| R3 (fish-1) | 20.34 | 34.23 | 36.46 | 39.45 |
| R3 (fish-2) | 22.92 | 31.19 | 34.98 | 37.65 |
| R3 (fish-3) | 19.62 | 32.32 | 34.34 | 40.35 |
| Average | 20.96 | 32.58 | 35.26 | 39.15 |

| 4. Blood was collected from 3 fish per replication (9 fish/group/timepoint) | **Groups** | | | |
| --- | --- | --- | --- | --- |
| Sampling time: **96 hours post-infection with *S. iniae*** | **Blood parameter (Haematocrit%)** | | | |
| Replication | BS | BF | MS | Unvaccinated |
| R1 (fish-1) | 10.28 | 15.34 | 18.32 | 18.15 |
| R1 (fish-2) | 11.46 | 17.45 | 21.11 | 17.87 |
| R1 (fish-3) | 9.34 | 14.25 | 16.34 | 20.23 |
| Average | 10.36 | 15.68 | 18.59 | 18.75 |
|  |  |  |  |  |
| R2 (fish-1) | 16.43 | 23.23 | 23.45 | 24.42 |
| R2 (fish-2) | 14.66 | 21.15 | 19.48 | 25.94 |
| R2 (fish-3) | 15.17 | 19.34 | 18.45 | 21.49 |
| Average | 15.42 | 21.24 | 20.46 | 23.95 |
|  |  |  |  |  |
| R3 (fish-1) | 15.34 | 18.45 | 19.54 | 19.65 |
| R3 (fish-2) | 13.23 | 16.47 | 21.87 | 23.45 |
| R3 (fish-3) | 15.14 | 20.34 | 23.75 | 18.34 |
| Average | 14.57 | 18.42 | 21.72 | 20.48 |

| 4. Blood was collected from 3 fish per replication (9 fish/group/timepoint) | **Groups** | | | |
| --- | --- | --- | --- | --- |
| Sampling time: **96 hours post-infection with *S. iniae*** | **Immune parameter (Lysozyme, unit/ml)** | | | |
| Replication | BS | BF | MS | Unvaccinated |
| R1 (fish-1) | 25.34 | 35.69 | 42.22 | 38.24 |
| R1 (fish-2) | 23.87 | 32.56 | 40.15 | 36.12 |
| R1 (fish-3) | 22.34 | 35.43 | 39.34 | 38.05 |
| Average | 23.85 | 34.56 | 40.57 | 37.47 |
|  |  |  |  |  |
| R2 (fish-1) | 25.36 | 32.52 | 36.47 | 45.54 |
| R2 (fish-2) | 23.57 | 28.85 | 33.39 | 41.68 |
| R2 (fish-3) | 25.32 | 30.34 | 36.43 | 42.56 |
| Average | 24.75 | 30.57 | 35.43 | 43.26 |
|  |  |  |  |  |
| R3 (fish-1) | 21.23 | 33.44 | 44.23 | 41.23 |
| R3 (fish-2) | 23.56 | 35.47 | 47.09 | 41.09 |
| R3 (fish-3) | 22.89 | 32.34 | 38.49 | 38.19 |
| Average | 22.56 | 33.75 | 43.27 | 40.17 |

| 4. Blood was collected from 3 fish per replication (9 fish/group/timepoint) | **Groups** | | | |
| --- | --- | --- | --- | --- |
| Sampling time: **96 hours post-infection with *S. iniae*** | **Immune parameter (Phagocytosis%)** | | | |
| Replication | BS | BF | MS | Unvaccinated |
| R1 (fish-1) | 34.23 | 35.53 | 53.14 | 45.34 |
| R1 (fish-2) | 33.36 | 38.65 | 51.34 | 44.95 |
| R1 (fish-3) | 30.12 | 37.21 | 52.24 | 43.72 |
| Average | 32.57 | 37.13 | 52.24 | 44.67 |
|  |  |  |  |  |
| R2 (fish-1) | 20.36 | 39.56 | 49.33 | 42.23 |
| R2 (fish-2) | 22.22 | 37.79 | 45.28 | 40.41 |
| R2 (fish-3) | 19.34 | 41.57 | 47.23 | 38.41 |
| Average | 20.64 | 39.64 | 47.28 | 40.35 |
|  |  |  |  |  |
| R3 (fish-1) | 31.22 | 31.33 | 42.34 | 49.34 |
| R3 (fish-2) | 28.11 | 31.52 | 41.21 | 47.16 |
| R3 (fish-3) | 28.12 | 35.34 | 46.23 | 48.46 |
| Average | 29.15 | 32.73 | 43.26 | 48.32 |

| 4. Blood was collected from 3 fish per replication (9 fish/group/timepoint) | **Groups** | | | |
| --- | --- | --- | --- | --- |
| Sampling time: **96 hours post-infection with *S. iniae*** | **Immune parameter (Antibody level)** | | | |
| Replication | BS | BF | MS | Unvaccinated |
| R1 (fish-1) | 0.29 | 0.56 | 0.93 | 0.67 |
| R1 (fish-2) | 0.27 | 0.54 | 0.94 | 0.72 |
| R1 (fish-3) | 0.34 | 0.49 | 0.89 | 0.68 |
| Average | 0.3 | 0.53 | 0.92 | 0.69 |
|  |  |  |  |  |
| R2 (fish-1) | 0.32 | 0.87 | 0.89 | 0.78 |
| R2 (fish-2) | 0.35 | 0.61 | 0.97 | 0.62 |
| R2 (fish-3) | 0.29 | 0.74 | 0.78 | 0.85 |
| Average | 0.32 | 0.74 | 0.88 | 0.75 |
|  |  |  |  |  |
| R3 (fish-1) | 0.29 | 0.62 | 0.75 | 0.73 |
| R3 (fish-2) | 0.31 | 0.62 | 0.81 | 0.69 |
| R3 (fish-3) | 0.27 | 0.56 | 0.78 | 0.71 |
| Average | 0.29 | 0.6 | 0.78 | 0.71 |

**Note:** Bivalent Spray (BS); Bivalent Formulate (BF); Monovalent *S. iniae* (MS); Monovalent *A. hydrophila* (MA). Data were presented in main manuscript **Tables 3 and 4.**

**Sampling time:** **96 hours post-infection with *A. hydrophila***

| 5. Blood was collected from 3 fish per replication (9 fish/group/timepoint) | **Groups** | | | |
| --- | --- | --- | --- | --- |
| Sampling time: **96 hours post-infection with *A. hydrophila*** | **Blood parameter (Erythrocytes, 10^6/µl)** | | | |
| Replication | BS | BF | MS | Unvaccinated |
| R1 (fish-1) | 1.33 | 3.56 | 6.52 | 3.23 |
| R1 (fish-2) | 1.77 | 4.89 | 7.85 | 2.98 |
| R1 (fish-3) | 1.28 | 5.56 | 5.67 | 4.71 |
| Average | 1.46 | 4.67 | 6.68 | 3.64 |
|  |  |  |  |  |
| R2 (fish-1) | 2.33 | 6.34 | 6.45 | 6.43 |
| R2 (fish-2) | 3.47 | 4.63 | 5.55 | 7.82 |
| R2 (fish-3) | 2.78 | 5.41 | 4.41 | 6.39 |
| Average | 2.86 | 5.46 | 5.47 | 6.88 |
|  |  |  |  |  |
| R3 (fish-1) | 3.23 | 3.55 | 6.46 | 5.55 |
| R3 (fish-2) | 2.58 | 3.91 | 5.39 | 4.62 |
| R3 (fish-3) | 2.44 | 2.98 | 4.89 | 6.54 |
| Average | 2.75 | 3.48 | 5.58 | 5.57 |

| 5. Blood was collected from 3 fish per replication (9 fish/group/timepoint) | **Groups** | | | |
| --- | --- | --- | --- | --- |
| Sampling time: **96 hours post-infection with *A. hydrophila*** | **Blood parameter (Thrombocytes, 10^3/µl)** | | | |
| Replication | BS | BF | MS | Unvaccinated |
| R1 (fish-1) | 15.34 | 22.22 | 19.32 | 19.48 |
| R1 (fish-2) | 15.22 | 19.62 | 20.34 | 16.12 |
| R1 (fish-3) | 12.25 | 19.24 | 18.45 | 19.45 |
| Average | 14.27 | 20.36 | 19.37 | 18.35 |
|  |  |  |  |  |
| R2 (fish-1) | 13.32 | 15.39 | 23.45 | 14.59 |
| R2 (fish-2) | 16.45 | 13.49 | 21.27 | 19.94 |
| R2 (fish-3) | 17.42 | 13.36 | 22.54 | 14.85 |
| Average | 15.73 | 14.08 | 22.42 | 16.46 |
|  |  |  |  |  |
| R3 (fish-1) | 14.34 | 23.45 | 15.45 | 27.45 |
| R3 (fish-2) | 12.73 | 22.19 | 18.25 | 24.77 |
| R3 (fish-3) | 10.31 | 21.14 | 15.32 | 24.52 |
| Average | 12.46 | 22.26 | 16.34 | 25.58 |

| 5. Blood was collected from 3 fish per replication (9 fish/group/timepoint) | **Groups** | | | |
| --- | --- | --- | --- | --- |
| Sampling time: **96 hours post-infection with *A. hydrophila*** | **Blood parameter (Leucocytes, 10^3/µl)** | | | |
| Replication | BS | BF | MS | Unvaccinated |
| R1 (fish-1) | 23.18 | 23.45 | 27.74 | 28.61 |
| R1 (fish-2) | 21.53 | 22.52 | 24.98 | 25.95 |
| R1 (fish-3) | 19.34 | 22.94 | 27.77 | 27.85 |
| Average | 21.35 | 22.97 | 26.83 | 27.47 |
|  |  |  |  |  |
| R2 (fish-1) | 20.33 | 25.45 | 32.34 | 25.45 |
| R2 (fish-2) | 21.44 | 21.37 | 28.01 | 24.21 |
| R2 (fish-3) | 19.31 | 23.23 | 29.92 | 23.42 |
| Average | 20.36 | 23.35 | 30.09 | 24.36 |
|  |  |  |  |  |
| R3 (fish-1) | 19.34 | 27.34 | 28.44 | 25.34 |
| R3 (fish-2) | 16.13 | 26.82 | 27.72 | 26.62 |
| R3 (fish-3) | 17.27 | 22.22 | 25.95 | 25.95 |
| Average | 17.58 | 25.46 | 27.37 | 25.97 |

| 5. Blood was collected from 3 fish per replication (9 fish/group/timepoint) | **Groups** | | | |
| --- | --- | --- | --- | --- |
| Sampling time: **96 hours post-infection with A. hydrophila** | **Blood parameter (Lymphocytes, 10^3/µl)** | | | |
| Replication | BS | BF | MS | Unvaccinated |
| R1 (fish-1) | 21.87 | 29.45 | 34.36 | 30.45 |
| R1 (fish-2) | 22.63 | 26.52 | 33.41 | 32.59 |
| R1 (fish-3) | 18.44 | 29.41 | 30.48 | 31.34 |
| Average | 20.98 | 28.46 | 32.75 | 31.46 |
|  |  |  |  |  |
| R2 (fish-1) | 29.64 | 32.34 | 28.44 | 30.23 |
| R2 (fish-2) | 28.32 | 30.87 | 28.55 | 30.81 |
| R2 (fish-3) | 27.45 | 29.58 | 31.39 | 27.61 |
| Average | 28.47 | 30.93 | 29.46 | 29.55 |
|  |  |  |  |  |
| R3 (fish-1) | 20.34 | 29.44 | 31.83 | 27.12 |
| R3 (fish-2) | 22.25 | 28.71 | 28.97 | 26.07 |
| R3 (fish-3) | 19.33 | 29.63 | 27.25 | 25.53 |
| Average | 20.64 | 29.26 | 29.35 | 26.24 |

| 5. Blood was collected from 3 fish per replication (9 fish/group/timepoint) | **Groups** | | | |
| --- | --- | --- | --- | --- |
| Sampling time: **96 hours post-infection with *A. hydrophila*** | **Blood parameter (Monocytes, 10^3/µl)** | | | |
| Replication | BS | BF | MS | Unvaccinated |
| R1 (fish-1) | 0.22 | 0.34 | 0.43 | 0.32 |
| R1 (fish-2) | 0.26 | 0.34 | 0.24 | 0.33 |
| R1 (fish-3) | 0.21 | 0.31 | 0.38 | 0.31 |
| Average | 0.23 | 0.33 | 0.35 | 0.32 |
|  |  |  |  |  |
| R2 (fish-1) | 0.29 | 0.29 | 0.43 | 0.36 |
| R2 (fish-2) | 0.26 | 0.31 | 0.31 | 0.38 |
| R2 (fish-3) | 0.23 | 0.24 | 0.28 | 0.31 |
| Average | 0.26 | 0.28 | 0.34 | 0.35 |
|  |  |  |  |  |
| R3 (fish-1) | 0.29 | 0.34 | 0.32 | 0.33 |
| R3 (fish-2) | 0.25 | 0.33 | 0.28 | 0.35 |
| R3 (fish-3) | 0.33 | 0.29 | 0.33 | 0.37 |
| Average | 0.29 | 0.32 | 0.31 | 0.35 |

| 5. Blood was collected from 3 fish per replication (9 fish/group/timepoint) | **Groups** | | | |
| --- | --- | --- | --- | --- |
| Sampling time: 96 hours post-infection with *A. hydrophila* | **Blood parameter (Granulocytes, 10^3/µl)** | | | |
| Replication | BS | BF | MS | Unvaccinated |
| R1 (fish-1) | 3.22 | 4.34 | 6.44 | 7.45 |
| R1 (fish-2) | 2.61 | 5.34 | 6.58 | 6.47 |
| R1 (fish-3) | 2.12 | 4.33 | 4.56 | 5.67 |
| Average | 2.65 | 4.67 | 5.86 | 6.53 |
|  |  |  |  |  |
| R2 (fish-1) | 2.35 | 5.45 | 6.42 | 6.49 |
| R2 (fish-2) | 1.83 | 6.44 | 5.35 | 4.53 |
| R2 (fish-3) | 1.94 | 5.39 | 4.67 | 7.61 |
| Average | 2.04 | 5.76 | 5.48 | 6.21 |
|  |  |  |  |  |
| R3 (fish-1) | 4.78 | 6.47 | 8.36 | 5.23 |
| R3 (fish-2) | 3.97 | 5.95 | 7.17 | 5.34 |
| R3 (fish-3) | 3.79 | 5.49 | 5.35 | 5.12 |
| Average | 4.18 | 5.97 | 6.96 | 5.23 |

| 5. Blood was collected from 3 fish per replication (9 fish/group/timepoint) | **Groups** | | | |
| --- | --- | --- | --- | --- |
| Sampling time: 96 hours post-infection with *A. hydrophila* | **Blood parameter (Haemoglobin, g/dl)** | | | |
| Replication | BS | BF | MS | Unvaccinated |
| R1 (fish-1) | 3.25 | 5.44 | 6.54 | 6.46 |
| R1 (fish-2) | 3.52 | 6.63 | 4.82 | 5.29 |
| R1 (fish-3) | 2.98 | 4.34 | 5.53 | 4.57 |
| Average | 3.25 | 5.47 | 5.63 | 5.44 |
|  |  |  |  |  |
| R2 (fish-1) | 3.45 | 6.45 | 6.43 | 7.18 |
| R2 (fish-2) | 3.29 | 5.47 | 7.31 | 5.35 |
| R2 (fish-3) | 2.98 | 5.45 | 5.52 | 4.54 |
| Average | 3.24 | 5.79 | 6.42 | 5.69 |
|  |  |  |  |  |
| R3 (fish-1) | 5.14 | 6.32 | 7.45 | 5.44 |
| R3 (fish-2) | 4.34 | 7.34 | 8.78 | 7.33 |
| R3 (fish-3) | 3.48 | 5.33 | 6.45 | 7.42 |
| Average | 4.32 | 6.33 | 7.56 | 6.73 |

| 5. Blood was collected from 3 fish per replication (9 fish/group/timepoint) | **Groups** | | | |
| --- | --- | --- | --- | --- |
| Sampling time: **96 hours post-infection with *A. hydrophila*** | **Blood parameter (MCH, pg)** | | | |
| Replication | BS | BF | MS | Unvaccinated |
| R1 (fish-1) | 15.34 | 15.34 | 15.76 | 12.43 |
| R1 (fish-2) | 14.61 | 13.27 | 12.72 | 15.74 |
| R1 (fish-3) | 14.33 | 14.35 | 15.47 | 12.48 |
| Average | 14.76 | 14.32 | 14.65 | 13.55 |
|  |  |  |  |  |
| R2 (fish-1) | 15.37 | 15.67 | 18.84 | 15.56 |
| R2 (fish-2) | 17.83 | 16.82 | 17.45 | 14.36 |
| R2 (fish-3) | 14.56 | 14.55 | 16.72 | 14.45 |
| Average | 15.92 | 15.68 | 17.67 | 14.79 |
|  |  |  |  |  |
| R3 (fish-1) | 15.34 | 14.22 | 13.24 | 16.34 |
| R3 (fish-2) | 14.81 | 13.32 | 15.69 | 18.13 |
| R3 (fish-3) | 14.37 | 15.21 | 12.35 | 15.27 |
| Average | 14.84 | 14.25 | 13.76 | 16.58 |

| 5. Blood was collected from 3 fish per replication (9 fish/group/timepoint) | **Groups** | | | |
| --- | --- | --- | --- | --- |
| Sampling time: 96 hours post-infection with *A. hydrophila* | **Blood parameter (MCHC, g/dl)** | | | |
| Replication | BS | BF | MS | Unvaccinated |
| R1 (fish-1) | 23.87 | 21.23 | 19.33 | 24.23 |
| R1 (fish-2) | 20.75 | 20.29 | 17.52 | 24.31 |
| R1 (fish-3) | 21.23 | 19.62 | 21.26 | 24.33 |
| Average | 21.95 | 20.38 | 19.37 | 24.29 |
|  |  |  |  |  |
| R2 (fish-1) | 18.22 | 22.24 | 22.23 | 19.62 |
| R2 (fish-2) | 21.18 | 22.39 | 23.72 | 18.44 |
| R2 (fish-3) | 19.04 | 20.14 | 21.31 | 19.45 |
| Average | 19.48 | 21.59 | 22.42 | 19.17 |
|  |  |  |  |  |
| R3 (fish-1) | 19.34 | 27.75 | 14.62 | 23.82 |
| R3 (fish-2) | 20.23 | 28.12 | 16.78 | 23.98 |
| R3 (fish-3) | 16.14 | 26.54 | 17.62 | 25.82 |
| Average | 18.57 | 27.47 | 16.34 | 24.54 |

| 5. Blood was collected from 3 fish per replication (9 fish/group/timepoint) | **Groups** | | | |
| --- | --- | --- | --- | --- |
| Sampling time: 96 hours post-infection with *A. hydrophila* | **Blood parameter (Haematocrit%)** | | | |
| Replication | BS | BF | MS | Unvaccinated |
| R1 (fish-1) | 12.11 | 17.45 | 19.34 | 20.34 |
| R1 (fish-2) | 11.33 | 19.25 | 18.43 | 17.96 |
| R1 (fish-3) | 10.34 | 18.38 | 17.34 | 16.33 |
| Average | 11.26 | 18.36 | 18.37 | 18.21 |
|  |  |  |  |  |
| R2 (fish-1) | 14.34 | 19.34 | 21.42 | 14.33 |
| R2 (fish-2) | 14.63 | 19.25 | 20.85 | 13.67 |
| R2 (fish-3) | 12.49 | 19.58 | 19.56 | 12.35 |
| Average | 13.82 | 19.39 | 20.61 | 13.45 |
|  |  |  |  |  |
| R3 (fish-1) | 14.32 | 19.44 | 18.34 | 19.32 |
| R3 (fish-2) | 13.66 | 16.86 | 21.41 | 17.71 |
| R3 (fish-3) | 12.46 | 15.84 | 18.39 | 17.33 |
| Average | 13.48 | 17.38 | 19.38 | 18.12 |

| 5. Blood was collected from 3 fish per replication (9 fish/group/timepoint) | **Groups** | | | |
| --- | --- | --- | --- | --- |
| Sampling time: **96 hours post-infection with *A. hydrophila*** | **Immune parameter (Lysozyme, unit/ml)** | | | |
| Replication | BS | BF | MS | Unvaccinated |
| R1 (fish-1) | 23.22 | 29.34 | 42.45 | 35.23 |
| R1 (fish-2) | 25.34 | 29.41 | 41.32 | 37.23 |
| R1 (fish-3) | 24.55 | 27.44 | 40.34 | 36.62 |
| Average | 24.37 | 28.73 | 41.37 | 36.36 |
|  |  |  |  |  |
| R2 (fish-1) | 28.33 | 34.56 | 49.34 | 45.42 |
| R2 (fish-2) | 26.18 | 36.28 | 48.23 | 45.04 |
| R2 (fish-3) | 25.23 | 35.54 | 47.27 | 45.32 |
| Average | 26.58 | 35.46 | 48.28 | 45.26 |
|  |  |  |  |  |
| R3 (fish-1) | 32.57 | 44.34 | 40.57 | 38.23 |
| R3 (fish-2) | 34.31 | 40.73 | 38.69 | 32.45 |
| R3 (fish-3) | 30.83 | 42.19 | 39.45 | 35.76 |
| Average | 32.57 | 42.42 | 39.57 | 35.48 |

| 5. Blood was collected from 3 fish per replication (9 fish/group/timepoint) | **Groups** | | | |
| --- | --- | --- | --- | --- |
| Sampling time: **96 hours post-infection with *A. hydrophila*** | **Immune parameter (Phagocytosis%)** | | | |
| Replication | BS | BF | MS | Unvaccinated |
| R1 (fish-1) | 29.54 | 32.34 | 56.43 | 57.34 |
| R1 (fish-2) | 29.81 | 35.03 | 53.12 | 53.72 |
| R1 (fish-3) | 32.33 | 36.31 | 56.23 | 54.72 |
| Average | 30.56 | 34.56 | 55.26 | 55.26 |
|  |  |  |  |  |
| R2 (fish-1) | 19.34 | 42.34 | 49.34 | 46.45 |
| R2 (fish-2) | 19.73 | 39.43 | 44.12 | 47.03 |
| R2 (fish-3) | 21.41 | 39.34 | 46.25 | 42.33 |
| Average | 20.16 | 40.37 | 46.57 | 45.27 |
|  |  |  |  |  |
| R3 (fish-1) | 27.34 | 37.23 | 43.23 | 42.34 |
| R3 (fish-2) | 30.56 | 36.85 | 41.16 | 40.27 |
| R3 (fish-3) | 27.45 | 35.48 | 42.45 | 39.46 |
| Average | 28.45 | 36.52 | 42.28 | 40.69 |

| 5. Blood was collected from 3 fish per replication (9 fish/group/timepoint) | **Groups** | | | |
| --- | --- | --- | --- | --- |
| Sampling time: **96 hours post-infection with *A. hydrophila*** | **Blood parameter (Antibody level)** | | | |
| Replication | BS | BF | MS | Unvaccinated |
| R1 (fish-1) | 0.31 | 0.43 | 0.84 | 0.73 |
| R1 (fish-2) | 0.34 | 0.47 | 0.77 | 0.76 |
| R1 (fish-3) | 0.28 | 0.54 | 0.76 | 0.67 |
| Average | 0.31 | 0.48 | 0.79 | 0.72 |
|  |  |  |  |  |
| R2 (fish-1) | 0.27 | 0.57 | 0.72 | 0.71 |
| R2 (fish-2) | 0.29 | 0.42 | 0.68 | 0.68 |
| R2 (fish-3) | 0.31 | 0.63 | 0.73 | 0.65 |
| Average | 0.29 | 0.54 | 0.71 | 0.68 |
|  |  |  |  |  |
| R3 (fish-1) | 0.32 | 0.67 | 0.84 | 0.61 |
| R3 (fish-2) | 0.34 | 0.73 | 0.68 | 0.77 |
| R3 (fish-3) | 0.27 | 0.67 | 0.76 | 0.69 |
| Average | 0.31 | 0.69 | 0.76 | 0.69 |

**Note:** Bivalent Spray (BS); Bivalent Formulate (BF); Monovalent *S. iniae* (MS); Monovalent *A. hydrophila* (MA). Data were presented in main manuscript **Tables 5 and 6.**
